# Toward a continuum description of lubrication in highly pressurized nanometer-wide constrictions: The importance of accurate slip laws

**DOI:** 10.1126/sciadv.adi2649

**Published:** 2023-12-01

**Authors:** Andrea Codrignani, Stefan Peeters, Hannes Holey, Franziska Stief, Daniele Savio, Lars Pastewka, Gianpietro Moras, Kerstin Falk, Michael Moseler

**Affiliations:** ^1^ Microtribology Center μTC, Fraunhofer Institute for Mechanics of Materials IWM, Wöhlerstraße 11, 79108 Freiburg, Germany.; ^2^ Freiburg Materials Research Center, University of Freiburg, Stefan-Meier-Straße 21, 79104 Freiburg, Germany.; ^3^ Institute of Physics, University of Freiburg, Hermann-Herder-Straße 3a, 79104 Freiburg, Germany.; ^4^ Freudenberg Technology Innovation SE & Co. KG, Höhnerweg 2-4, 69469 Weinheim, Germany.; ^5^ Department of Microsystems Engineering, University of Freiburg, Georges-Köhler-Allee 103, 79110 Freiburg, Germany.

## Abstract

The Reynolds lubrication equation (RLE) is widely used to design sliding contacts in mechanical machinery. While providing an excellent description of hydrodynamic lubrication, friction in boundary lubrication regions is usually considered by empirical laws, because continuum theories are expected to fail for lubricant film heights *h*_0_ ≪ 10 nm, especially in highly loaded tribosystems with normal pressures *p*_n_ ≫ 0.1 GPa. Here, the performance of RLEs is validated by molecular dynamics simulations of pressurized (with *p*_n_ = 0.2 to 1 GPa) hexadecane in a gold converging-diverging channel with minimum gap heights *h*_0_ = 1.4 to 9.7 nm. For *p*_n_ ≤ 0.4 GPa and *h*_0_ ≥ 5 nm, agreement with the RLE requires accurate constitutive laws for pressure-dependent density and viscosity. An additional nonlinear wall slip law relating wall slip velocities to local shear stresses extends the RLE’s validity to even the most severe loading condition *p*_n_ = 1 GPa and *h*_0_ = 1.4 nm. Our results demonstrate an innovative route for continuum modeling of highly loaded tribological contacts under boundary lubrication.

## Teaser

Reynold’s lubrication equation extended by a wall slip law describes lubrication in highly pressurized nanometer constrictions.

## INTRODUCTION

Modern, compact, and efficient tribological systems are often operated in mixed lubrication or even boundary lubrication, meaning small gaps, frequently high pressures, and occasional solid-solid contacts between the lubricated sliding surfaces (*1*). In particular, the need for climate-friendly lubricants with low viscosities (*2*), an increase in assembly precision of lubricated contacts for electric vehicles (*3*), and high-performance coating techniques (*4*) that allow smaller assembly tolerances have driven the shift to operating devices in the mixed lubrication regime. Downsizing markedly increases loads in tribological components (often in the gigapascal range) resulting in an additional driving force toward boundary lubricated contacts (*5*). Under such severe conditions, the film thickness in typical applications can reach a few nanometers (*6*), becoming comparable to the size of the lubricant molecules themselves. At this scale, a current state-of-the-art continuum description of lubricant flow is expected to lose its validity due to density layering (*7*), solvation forces (*8*), the emergence of solid-like states (*9*, *10*), increased viscosities, or wall slip (*11*–*16*) [see also (*17*) for a comprehensive review of atomistic simulations of confined lubricant films].

The Reynolds lubrication equation (RLE) is the most commonly used continuum equation for flow calculations in lubricated systems (*18*). Although the RLE was proposed at the end of the 19th century for incompressible laminar Newtonian flows (*19*), the past decades have seen research in extending its applicability to lubricants exhibiting compressibility, piezoviscosity, shear thinning, and cavitation (*1*, *11*, *20*). These extensions have rendered the RLE a predictive description for elastohydrodynamic lubrication (EHL) of technically relevant tribocontacts (*20*), provided quantitative constitutive laws for compressibility and viscosity are used (*21*). In combination with empirical friction coefficients for boundary lubrication regions, the RLE is also used for mixed lubrication problems (*22*, *23*). Technically, the RLE is used for local gap heights exceeding a certain threshold (of the order of 0.1 to 1 μm) to obtain the hydrodynamic contribution to friction while smaller gaps are assumed to be in solid-solid contact and modeled via a Coulomb-Amontons friction law or a Bowden-Tabor (*24*) constant interfacial shear stress. The choice of this threshold is more a matter of convenience than of a physical reasoning. It would be very useful to explore the lower gap size limits for a continuum description of the lubricant flow—especially for high local pressures characteristic for EHL contacts. By extending the RLE realm to smaller scales, the importance of empirical solid-solid contact friction laws could be reduced and therefore the predictive power of mixed lubrication calculations would improve substantially.

**Fig. 1.**
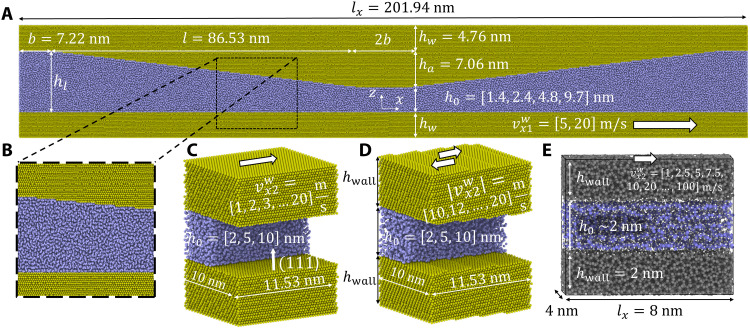
Atomistic models used in the MD simulations. (**A**) Converging-diverging channel of gold filled with hexadecane. Gold atoms are depicted in yellow and hexadecane molecules are depicted in blue. The *z*/*x* aspect axis ratio is 2.5 to improve the readability of the plot. Periodic boundaries are applied in *x* and *y* directions. (**B**) Magnification of the dashed rectangle in (A) showing the atomic structure of the gold surfaces. (**C** and **D**) Parallel channels with Au surfaces having the same roughness characteristics as the bottom and top walls of the CDC, namely, atomically flat Au(111) and Au(111) terraces, respectively. Only half of the hexadecane molecules are shown. (**E**) Parallel channel formed by hydrogenated amorphous carbon surfaces (in black) and filled with 1-decene trimers (in blue). Because of the pressure equilibration in the hexadecane and the elasticity of the walls of the CDC, there are deviations from the target values *h*_0_ = [2, 3, 5, 10] nm of the minimum gap height. Pressure equilibration was performed with *p*_n_ = [0.2, 0.4, 0.6, 0.8, 1] GPa in (A), *p*_n_ = [0.1, 0.4, 0.6, 0.8, 1] GPa in (C), *p*_n_ = [0.8, 1] GPa in (D), and *p*_n_ = [0.2, 0.5, 1, 1.5, 2] GPa in (E). During sliding with constant *h*_0_, there are small deviations from the nominal values of the average pressure (<10% of the values).

In the present work, isothermal nonequilibrium molecular dynamics (MD) simulations of hexadecane in a gold converging-diverging channel (CDC) (depicted in Fig. 1, A and B) are performed to generate realistic benchmark data representative of mineral oils lubricating an asperity contact between two metal surfaces. In a previous related study, some of the authors have used this alkane/gold model to study the onset of cavitation and its continuum description in a parallel channel with heterogeneous slip conditions at moderate pressures (*25*), while the current work addresses much higher pressures and a variation in the channel height. A profound MD characterization of our atomistic hexadecane lubricant model provides an equation of state ρ(*p*) as well as a pressure- and shear rate–dependent constitutive law for the viscosity $\eta (p,\dot{\gamma })$. With these data, the validity limit of the RLE description for gaps *h*_0_ in the single-digit nanometer range and pressures *p* approaching the gigapascal regime is explored. A failure of such a traditional RLE treatment for high pressures can be traced back to the violation of the no-slip boundary condition on the gold (111) facets in the CDC. By a separate parametric MD study of hexadecane in parallel gold channels (Fig. 1, C and D), the pressure dependence of wall slip is quantified, and the existence of a constitutive law *v*_s_ = *v*_s_(τ, *p*) that relates local shear stress τ at the wall with slip velocity *v*_s_ is demonstrated. A *v*_s_(τ, *p*) law is also found for technically relevant systems such as diamond-like carbon (DLC) channels filled with poly-α-olefin (PAO) lubricant (Fig. 1E). Last, we show that an extension of the RLE by *v*_s_ = *v*_s_(τ, *p*) results in a model that allows for a quantitative description of pressure and velocity profiles in our MD simulations of CDCs for minimum gap sizes and local pressure that are of the order of 1 nm and 1 GPa, respectively.

## RESULTS

To explore the validity of the Reynolds equation in the context of boundary lubrication applications, we consider a system at the edge of the applicability range of continuum methods. A main simulation campaign is designed based on a narrow CDC. Figure 1A shows the exact dimensions of the CDC whose geometry is described by the height function

$$h(x)=\left\{ \begin{array}{c} h_{0},|x|\;<b\\ h_{0}+\frac{h_{l}-h_{0}}{l}\;(|x|-\;b),b\leq \;|x|<l+b\\ h_{l},l+b\leq \;|x| \end{array}\right.$$ (1)

with the height *h_l_* of the channel at the simulation cell boundaries, the length *l* of the converging and diverging sections, the length 2*b* of the parallel section in the middle of the CDC, and the targeted central minimum gap heights *h*_0_ of 2, 3, 5, and 10 nm in our atomistic simulations. The function *h*(*x*) is used to carve the CDC out of an *l_x_* = 201.9 nm × *l_y_* = 5 nm × *l_z_* = 26.6 nm block of crystalline gold with (111) orientation in the *z* direction and periodic boundary conditions in *x* and *y*. This void region is filled with *n*-hexadecane molecules and pressurized to a target normal pressure *p*_n_ at a temperature *T* = 400 K. Various minimum gap heights *h*_0_ are established by adjusting the number of lubricant molecules in the channel. During pressure equilibration, the gap heights stabilize at *h*_0_ = 1.4, 2.4, 4.8, and 9.7 nm. Shear flow is induced by translating the bottommost gold atoms as a rigid layer at constant height with a velocity $v_{x1}^{w}$ in the *x* direction, while the topmost gold atoms are kept immobile (resulting in a wall velocity $v_{x2}^{w}=0$). The resulting flow in the hexadecane reaches a steady state after approximately 10 ns.

**Fig. 2.**
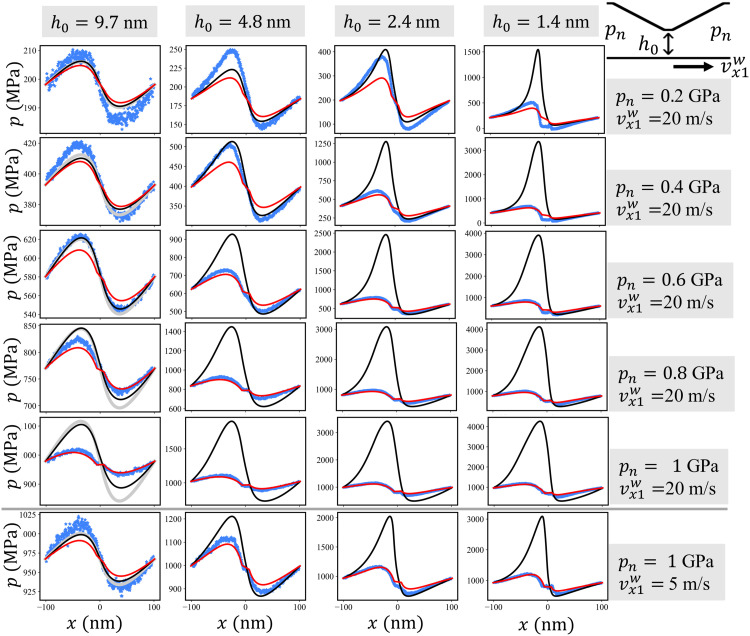
Pressure profile in the CDC. In the top five rows, *p*(*x*) is shown for $v_{x1}^{w}=20\;m/s$, minimum gap heights *h*_0_ = [9.7, 4.8, 2.4, and 1.4] nm, and normal pressures *p*_n_ = [0.2, 0.4, 0.6, 0.8, 1.0] GPa. The bottom row reports the pressure profile for $v_{x1}^{w}=5\;m/s$ and *p*_n_ = 1.0 GPa. Blue stars represent the pressure *p*(*x*) obtained from 10-ns steady-state averages over MD trajectories. Gray curves in the first column are the exact analytic solution for the incompressible isoviscous Reynolds equation. The numerical solutions of the Reynolds equation with either no-slip or slip boundary condition are displayed as black and red curves, respectively. See fig. S1 for all $v_{x1}^{w}=5\;m/s$ results and figs. S2 and S3 for the corresponding density profiles.

For an incompressible and isoviscous fluid in the CDC, the corresponding Reynolds equation

$$\frac{d}{dx}\left[\frac{h^{3}}{12\eta }\frac{dp}{dx}\right]=\frac{d}{dx}h\frac{v_{x1}^{w}}{2}$$ (2)

can be solved analytically by evaluating the integral

$$p(x)-p(0)=6\eta v_{x1}^{w}\int_{0}^{x}\left(\frac{1}{h{\left(x^{\prime }\right)}^{2}}-\frac{c}{h{\left(x^{\prime }\right)}^{3}}\right)dx^{\prime }$$ (3)

where η denotes the viscosity of the fluid at the respective *p*_n_ and *c* is an integration constant determined by the boundary condition *p*_n_ = *p*(*l* + 2*b*) = *p*(−*l* − 2*b*); see Materials and Methods for a detailed derivation of the analytical solution. Analytical pressure profiles are shown in Fig. 2 for a CDC with *h*(0) = *h*_0_ = 9.7 nm and normal pressures *p*_n_ = [0.2, 0.4, 0.6, 0.8, 1.0] GPa (see gray curves in the left most column of Fig. 2). As expected, there is a pressure increase in the converging section of the channel, followed by a drop in pressure in the diverging part. The prefactor $\eta v_{x1}^{w}$ in Eq. 3 indicates that the amplitude of this pressure oscillation grows with driving velocity and external pressure (because the constant viscosity η in Eq. 3 increases with the applied *p*_n_).

To compare our atomistic simulations with such a continuum solution, both wall surfaces are partitioned into *x*-bins with width Δ*x* = 0.4 nm, and 10-ns-long steady-state averages of the forces acting from the fluid on the gold atoms in the respective bin are recorded. Dividing the *z*- and *x*-force component through the bin area *l_x_*Δ*x* yields local pressures and frictional stresses, respectively. The resulting pressure profiles *p*(*x*) on the bottom wall are shown in Fig. 2 as blue stars. For the largest *h*_0_ = 9.7 nm and pressures *p*_n_ ≤ 0.6 GPa, satisfactory agreement between the atomistic *p*(*x*) and the analytic RLE solution is observed. However, an increase of the pressure to *p*_n_ = 1 GPa leads to a strong overestimation of the pressure variation along the CDC by the analytic solution (for both simulated velocities 20 and 5 m/s).

**Fig. 3.**
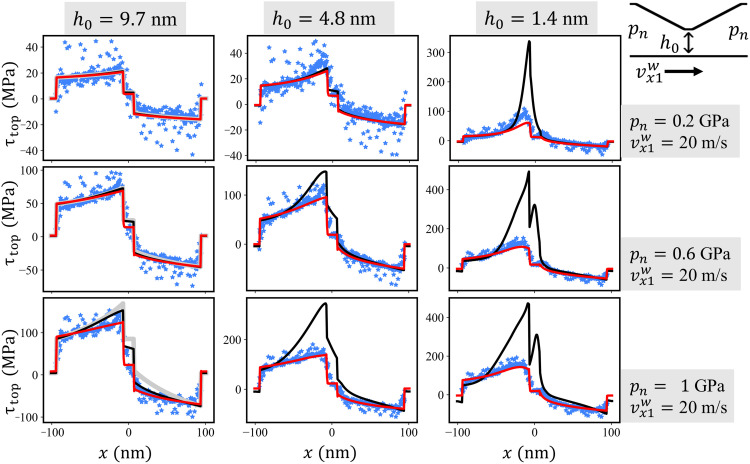
Frictional stress profile on the top wall of the CDC. Results for shear velocity $v_{x1}^{w}=20\;m/s$, minimum gap heights *h*_0_ = [9.7, 4.8, 1.4] nm, and normal pressures *p*_n_ = [0.2, 0.6, 1.0] GPa are shown. Blue dots represent the frictional stresses τ_top_(*x*) obtained from steady-state time averages of the local lateral forces (i.e., in the *x* direction) on the top wall atoms in the MD trajectories. Gray curves in the first column represent the exact analytic solution for the incompressible isoviscous Reynolds equation. The results of the numerical solution of the Reynolds equation with no-slip and slip boundary condition are displayed as black and red curves, respectively. See figs. S4 to S7 for the frictional stresses on the top and bottom walls of the full simulation campaign.

Furthermore, the analytic Reynolds solutions of the frictional stress profiles τ_top_(*x*) and τ_bot_(*x*) acting on the top and bottom walls, respectively, agree well with the MD profiles for *h*_0_ = 9.7 nm and *p*_n_ ≤ 0.6 GPa (Fig. 3 and figs. S4 to S7). Even for *h*_0_ = 9.7 nm and *p*_n_ = 1 GPa, the analytic τ_top_(*x*) is close to the MD result, while τ_bot_(*x*) deviates markedly from the atomistic benchmark (fig. S6). Because the frictional stress profiles τ_top_(*x*) on the tilted parts of the top wall includes additional contribution from the pressure profile

$$\tau_{\mathrm{top}}(x)=-\eta \frac{{\partial v}_{x}(x,z)}{\partial z}{|}_{z=h(x)}-p(x)\frac{dh(x)}{dx}$$ (4)

its agreement with MD is most likely a result of an error cancellation.

**Fig. 4.**
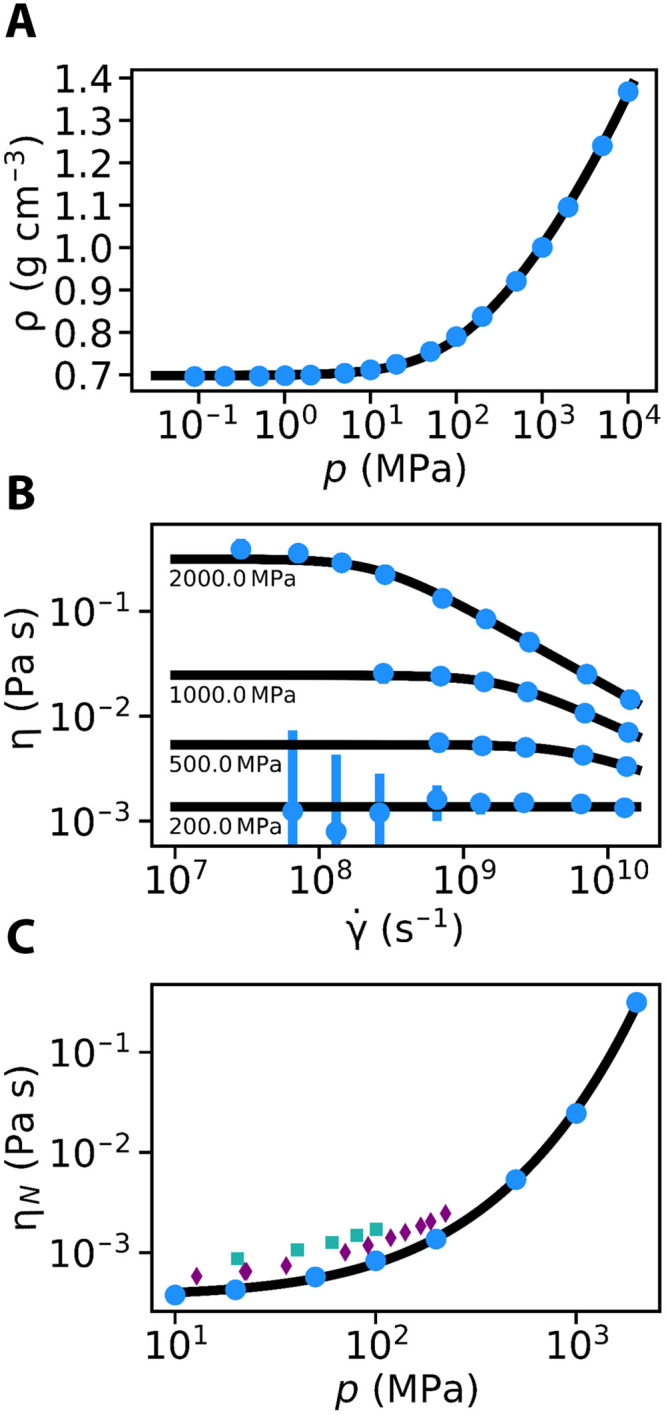
Calibration of the equation of state and the viscous constitutive law by MD. (**A**) Density-pressure relation of *n-*hexadecane at 400 K obtained from bulk equilibrium MD simulations (blue discs) and fit by the Tait-Murnaghan equation of state (black line). (**B**) Shear-thinning behavior of the hexadecane lubricant for pressures ranging from 0.2 to 2 GPa. Blue discs represent the viscosities obtained by nonequilibrium MD shearing simulations for various shear rates $\dot{\gamma }$. The MD data are fitted by the Carreau equation (black lines). (**C**) Dependence of the viscosity prefactor η*_N_* of the Carreau equation on pressure *p* (blue discs). The η*_N_*(*p*) values are fitted by the Roelands equation (black line). The purple diamonds and the green squares represent viscosity experiments at 429 K (*75*) and at 398 K (*76*), respectively.

Next, we investigate the validity and performance of a state-of-the-art continuum description of our atomistic benchmark system employing a no-slip Reynolds equation that takes into account compressibility and piezoviscosity via an equation of state for the density ρ(*p*) (*11*, *26*) and a constitutive law for the viscosity $\eta (p,\dot{\gamma })$, respectively. Here, $\dot{\gamma }$ denotes local shear rate and *p* denotes local pressure in the channels. In a first step, bulk equilibrium MD simulations of representative volume elements of the fluid were performed. A volume *V* with periodic boundaries was filled with *N* hexadecane molecules of molecular mass *M*, and the resulting hydrostatic pressure *p* was calculated. This provides data for the relationship of ρ = *NM*/*V* with *p* (blue discs in Fig. 4A). The compressibility is then described through the Tait-Murnaghan equation of state (*27*)

$$\rho (p)=\rho_{0}{\left[1+\frac{n_{\mathrm{TM}}}{K_{\mathrm{TM}}}\;(p-p_{\mathrm{TM}})\right]}^{\frac{1}{n_{\mathrm{TM}}}}$$ (5)

by fitting the parameters ρ_0_, *p*_TM_, *K*_TM_, and *n*_TM_ to the MD results. The values of the fit parameters are given in Materials and Methods. The ρ(*p*) in Eq. 5 is in excellent agreement with the atomistic results (see black solid line in Fig. 4A).

As a constitutive law for viscosity, the Carreau formula for shear thinning (*28*) is chosen

$$\eta (\dot{\gamma },p)=\eta_{N}(p){\left[1+{\left(\frac{\dot{\gamma }}{{\dot{\gamma }}_{0}(p)}\right)}^{2}\right]}^{\frac{n_{\mathrm{Car}}(p)-1}{2}}$$ (6)

and combined with the Roelands piezoviscosity formula (*29*)

$$\eta_{N}(p)=\eta_{0}\exp\left\{\ln\left(\frac{\eta_{0}}{\eta_{\infty }}\right)\left[-1+{\left(1+\frac{p}{p_{R}}\right)}^{z_{R}}\right]\right\}$$ (7)

for the Newtonian viscosity η*_N_*(*p*) in the Carreau equation. These constitutive laws are calibrated through nonequilibrium MD simulations of our pressurized lubricant volumes by applying shear flow boundary conditions with a shear rate $\dot{\gamma }$ (see details in Materials and Methods). Figure 4B depicts the dependence of viscosity η on $\dot{\gamma }$ for various pressures (blue discs). For each *p*, the parameters η*_N_*(*p*), ${\dot{\gamma }}_{0}(p)$, and *n*_Car_(*p*) are determined by fitting Eq. 6 to the MD results. The Carreau model provides a good description of our hexadecane model (black solid lines in Fig. 4B). In a subsequent step, Eq. 7 is fitted to the prefactors η*_N_*(*p*) (blue discs in Fig. 4C) by adjusting the parameters η_0_, *p_R_*, and *z_R_*. The Roelands equation describes this dependence very well (black solid line in Fig. 4C). Last, the remaining parameters ${\dot{\gamma }}_{0}(p)$ and *n*_Car_(*p*) in Eq. 6 are fitted with exponential functions ${\dot{\gamma }}_{0}(p)={\dot{\gamma }}_{00}\exp(-p/p_{{\dot{\gamma }}_{0}})$ and *n*_Car_(*p*) = *n*_0_exp(−*p*/*p*_n_Car__), and all fit results are combined to obtain a continuous function for $\eta (\dot{\gamma },p)$. All fitting parameters are provided in Materials and Methods. Note that the η*_N_*(*p*) of the used united atom force field (*29*) underestimates the experimental Newtonian viscosities (see diamonds and squares in Fig. 4C) by approximately 50% in agreement with (*30*).

After having established sound constitutive equations ρ(*p*) and $\eta (\dot{\gamma },p)$, we can now apply the compressible Reynolds equation

$$\frac{d}{dx}\left[\frac{\rho h^{3}}{12\eta }\frac{dp}{dx}\right]=\frac{d}{dx}\left[\rho h\frac{v_{x1}+v_{x2}}{2}\right]$$ (8)

to our CDC geometry *h*(*x*). Here, the velocities of the lubricant at the bottom and top wall are given by *v_x_*(*x*, 0) = *v*_*x*1_ and *v_x_*[*x*, *h*(*x*)] = *v*_*x*2_, respectively. Note that in this form, no assumptions have yet been made about the boundary conditions for the lubricant at the walls. First, we apply no-slip conditions by assuming that $v_{x2}=v_{x2}^{w}=0\;m/s$ and $v_{x1}=v_{x1}^{w}=20$ and 5 m/s.

The black solid lines in Fig. 2 show the pressure profiles obtained by a numerical solution of Eq. 8 with no-slip boundary conditions. A comparison with the MD simulations of the CDC (blue stars) leads to the conclusion that a compressible, piezoviscous Reynolds equation without wall slip shares the same deficiencies as the incompressible isoviscous analytic solution. It exhibits a reasonable agreement with the MD only as long as the gap height is large and the pressure small enough (compare blue stars with black lines for *h*_0 _≥ 5 nm and *p*_n_ ≤ 0.4 GPa in Fig. 2). For small gaps and high pressures, the continuum model severely overestimates the pressure variation *p*(*x*) along the sliding direction. A similar conclusion holds for the frictional stress τ_top_(*x*) and τ_bot_(*x*); see Fig. 3 and figs. S4 to S7.

**Fig. 5.**
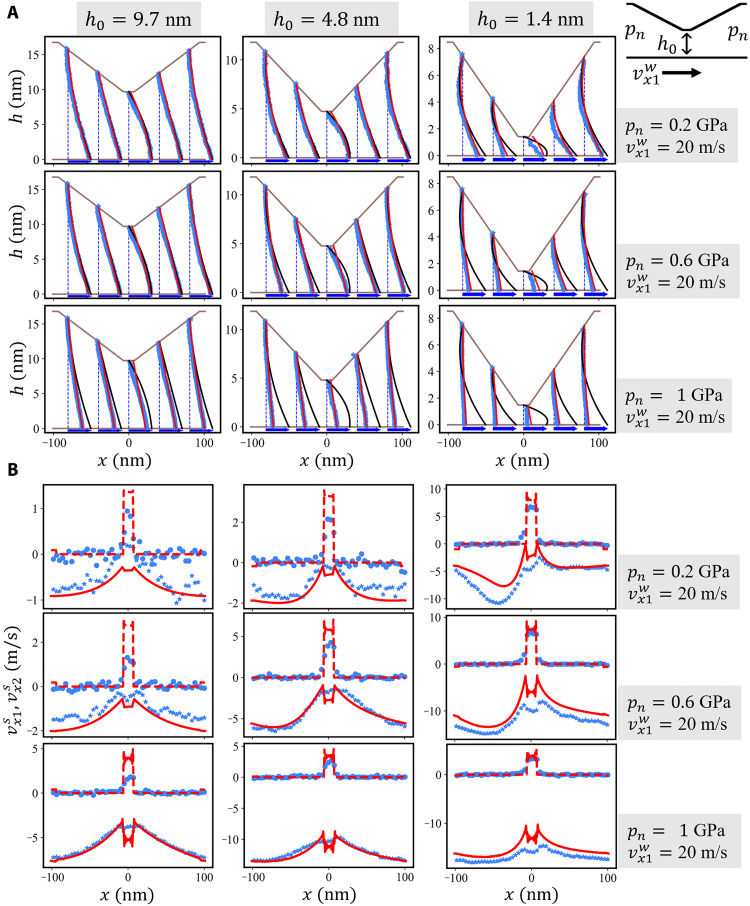
Velocity profiles of *n*-hexadecane in the CDC. (**A**) The results for the velocity profiles *v_x_*(*x*, *z*) at five different *x* positions are shown for a lower wall velocity of $v_{x1}^{w}=20\;m/s$ (depicted as blue arrows), minimum gap heights *h*_0_ = [9.7, 4.8, 1.4] nm (left to right), and normal pressures *p*_n_ = [0.2, 0.6, 1.0] GPa (top to bottom). Blue stars represent the data obtained from steady-state averages over MD trajectories. Results of the Reynolds equation with no-slip and slip boundary condition are displayed as black and red curves, respectively. (**B**) Corresponding slip velocity profiles. Blue discs and stars represent the slip velocity *v*_s_(*x*) at the top and bottom wall, respectively. The results of the Reynolds equation with slip boundary conditions are displayed as solid and dashed red curves for the bottom and top wall, respectively. See figs. S8 to S11 for the results of the full simulation campaign.

Next, a continuum velocity field *v_x_*(*x*, *z*) is computed from the atomistic trajectories by partitioning the computational domain in *x*-*z*-bins of the size 0.4 nm by 0.1 nm and performing 10-ns-long steady-state averages over the atomic velocities in the bins. The resulting *v_x_*(*x*, *z*) of the simulations with $v_{x1}^{w}=20\;m/s$ are shown in Fig. 5A. For each combination of *h*_0_ and *p*_n_, *v_x_*(*x*, *z*) is displayed for five different *x* values along the sliding direction (blue stars). The driving velocity $v_{x1}^{w}=20\;m/s$ of the lower wall is represented by blue arrows, which allows to identify violations of the no-slip boundary condition at the lower Au(111) wall of the CDC in certain cases. Alternatively, these cases can be identified in the slip velocity profiles $v_{x1}^{s}(x)$ and $v_{x2}^{s}(x)$ at the lower and upper wall, respectively (Fig. 5B). Especially at *p*_n_ = 1 GPa, slip velocities *v*_s_ ≫ 1 m/s occur approaching $v_{s}\approx v_{x1}^{w}$ in the most extreme case *h*_0_ = 1.4 nm. At this film thickness, molecular layering (*31*) and confinement-induced viscosity increases (*32*) have been noted experimentally for hexadecane.

Conversely, the hexadecane seems to slip less on the top wall. This is probably due to the more corrugated nanotopography of the tilted sections of the top wall (see zoom in Fig. 1B), where a staircase of Au(111) terraces has been created by cutting the (111) oriented gold crystal with a cutting plane tilted by an angle ϑ = 4.665°. Experiments with tetradecane show that the slip lengths decrease with increasing root mean square (RMS) roughness of polymer interfaces (*33*), and previous MD simulations indicate that, although boundary slip of strongly confined hexadecane occurs on atomically smooth gold surfaces, no-slip boundary conditions are recovered for randomly rough surfaces with RMS roughness of just 0.16 nm (*34*). We tried to estimate a critical length of the Au(111) terraces in our CDC that would reintroduce slip. Useful information comes from (*25*), where pentane was confined between smooth walls with sticking and slipping patches at moderate pressures (*p*_n_ = 250 MPa). In particular, the walls in (*25*) featured an Au(111) crystallographic orientation, with wall-fluid interactions similar to the present work in the sticking domains, and a 10-fold reduced interaction in the slipping ones. Figure 2B in (*25*) shows transition lengths of approximately 10 nm from no-slip to slip and vice versa. This provides a first guess of 20 nm for the critical terrace length to reintroduce slip in the system. Because the terraces in our CDC are one order of magnitude smaller slip is strongly suppressed.

**Fig. 6.**
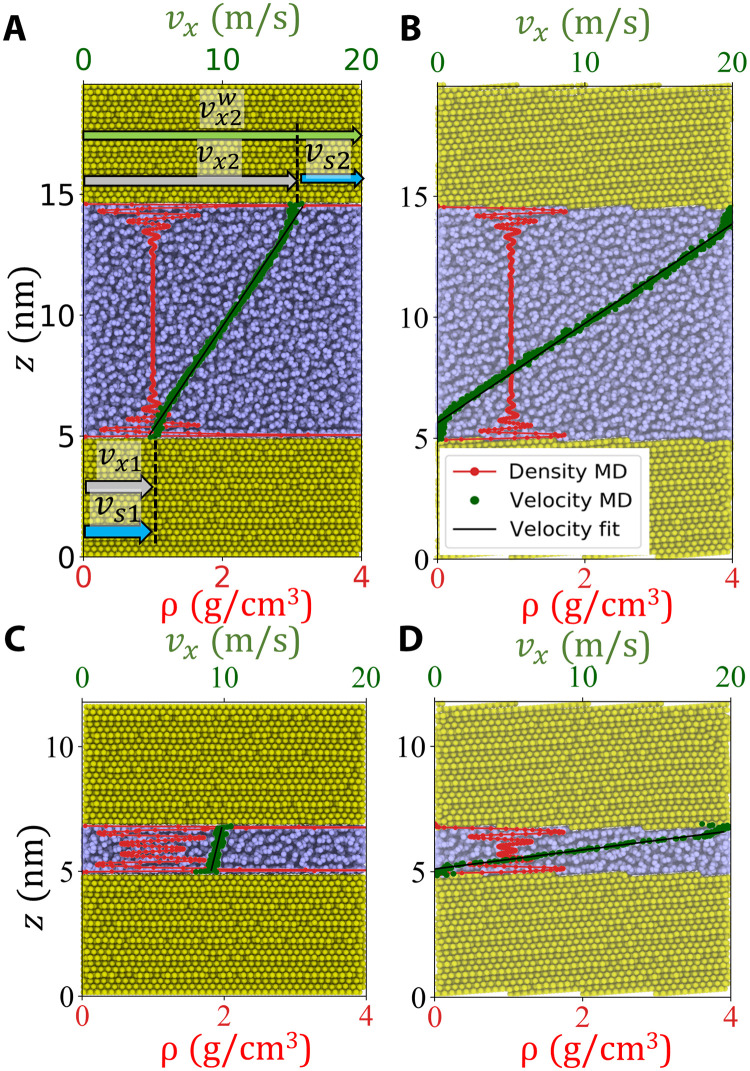
Velocity and density profiles in the parallel channel MD simulations. The time-averaged profiles are overlaid on top of atomistic snapshots to better show their position with respect to the wall. The displayed profiles were obtained at *p*_n_ = 1 GPa and with a wall velocity $v_{x2}^{w}=20\;m/s$. (**A** and **C**) represent the channel with atomically flat Au(111) walls, while (**B** and **D**) show the corresponding results for the terraced Au(111) surfaces. The nominal gap height is *h*_0_ = 10 nm in the top row of panels, while it is *h*_0_ = 2 nm in the bottom row.

To investigate the occurrence of slip on Au(111), we perform additional MD simulations in simpler setups. The shear flow of hexadecane confined by parallel gold walls is studied for two different gold surface structures: nominally flat Au(111) (Fig. 1C) and the staircase of Au(111) terraces (Fig. 1D). The latter has exactly the same roughness characteristic as the tilted sections of the top wall of our CDC (see Fig. 1B). In both studies, we measure the occurrence of slip at the fluid/wall interface in gaps with nominal heights ranging from 2 to 10 nm, a normal pressure ranging from 0.1 to 1 GPa, and top wall velocities $v_{x2}^{w}$ of up to 20 m/s [see Fig. 1 (C and D) for details]. This wide parameter space allows us to cover the transition from mild operating conditions, where the no-slip hypothesis still holds, to extreme conditions, where substantial slip occurs. More details on the MD setup are given in Materials and Methods. Representative density and velocity profiles of both parametric studies are displayed in Fig. 6 for *p*_n_ = 1 GPa and $v_{x2}^{w}=20\;m/s$.

Figure 6A shows a snapshot of the hexadecane molecules in a 10-nm-wide parallel channel between two Au(111) surfaces. The superimposed density profile ρ(*z*) displays the well-known oscillations in the vicinity of the walls (red curve) and a constant density of ρ(*z*) ≈ 1 g cm^−3^ in the bulk of the lubricant (7 nm ≤ *z* ≤ 13 nm). The steady-state velocity profile *v_x_*(*z*) is represented by a green solid line. A linear *v_x_*(*z*) indicates an expected Couette-type flow in the channel. Instead of approaching the zero velocity of the lower wall, the fluid velocity extrapolated to the lower Au(111) surface is finite $v_{x}(z=5\;\mathrm{nm})=v_{x1}^{s}=4\;m/s$. The same applies to the upper wall, where $v_{x}(z=15\;\mathrm{nm})=v_{x2}^{w}-v_{x2}^{s}=16\;m/s$. The overall slip velocity is computed as the average value from both walls, i.e., $v_{s}=(v_{x1}^{s}+v_{x2}^{s})/2=4\;m/s$. Under the same conditions, ρ(*z*) and *v_x_*(*z*) in the channel with the tilted Au(111) surface that exhibits staircase-like surface structures display a similar behavior (Fig. 6B) with only one exception—the absence of slip, i.e., *v*_s_ ≈ 0. It even seems that the last fluid layers at both walls move with the wall velocity such that the extrapolation of the linear section of *v_x_*(*z*) would yield a slightly negative *v*_s_. The lack of slip on Au(111) staircases can also be seen in the velocity profiles of Fig. 5 in the converging or diverging sections of the CDC geometry. Even if we decrease the height of the parallel channel to *h*_0_ = 2 nm, no slip is perceptible and still a clear Couette profile forms (green curve in Fig. 6D) despite the strong fluid layering covering the complete channel height; the red curve in Fig. 6D shows oscillations of ρ(*z*) all over the channel.

Note that we observed *v*_s_ ≈ 0 on terraced Au(111) for all simulated gap heights *h*_0_, pressures *p*_n_, and wall velocities $v_{x2}^{w}$, and therefore, the assumption of no-slip boundary conditions is well justified even for extremely narrow channels. This is in stark contrast to the *h*_0_ = 2 nm channel with flat Au(111) surfaces where the fluid flows with almost constant velocity $v_{x}(z)\approx v_{x2}^{w}/2$ (Fig. 6C) resulting in $v_{s}\approx v_{x2}^{w}/2$.

**Fig. 7.**
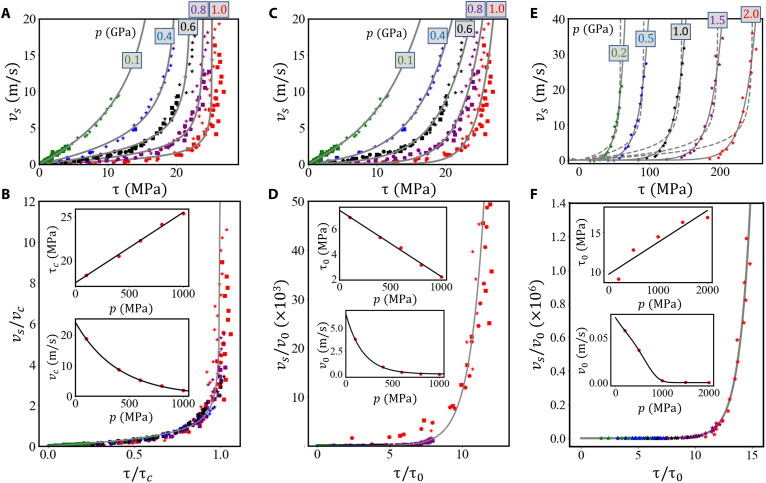
Wall slip velocity as a function of shear stress in the parallel channel with Au(111) surfaces. Each point corresponds to one MD shearing simulation with a fixed parameter set $\left(h_{0},p_{n},v_{x2}^{w}\right)$. The shape of the markers represents the gap height *h*_0_ (stars: 2 nm, squares: 5 nm, discs: 10 nm), while the colors distinguish the reference pressures *p* in the atomistic simulations. (**A**) Results *v*_s_(τ) for all considered parameter sets $\left(h_{0},p_{n},v_{x2}^{w}\right)$. The lines are fits of the constitutive slip law (Eq. 12). (**B**) Master curve for the slip velocity versus the shear stress obtained through normalization of the data in (A) by the characteristic velocity *v*_c_(*p*) and the limiting shear stress τ_c_(*p*), respectively. The gray line represents the dimensionless version of the constitutive law. Fit parameters τ_c_(*p*) and *v*_c_(*p*) are shown in the insets as red discs, while black lines are fit curves according to Eqs. 13 and 14. (**C**) Fit of the MD data in (A) using the Eyring model (Eq. 15). (**D**) Master curve obtained through normalization of the data in (C) by the characteristic velocity *v*_0_(*p*) and shear stress τ_0_(*p*), which were fitted according to Eqs. 16 and 17. (**E**) Fits of the constitutive slip laws for the parallel DLC channels lubricated by 1-decene trimers. The solid and dashed gray lines represent the fit with Eqs. 12 and 15, respectively. (**F**) Master curve obtained from the normalization of the data in (E) using Eq. 15, with the insets showing the fit of *v*_0_(*p*) and τ_0_(*p*) with Eqs. 16 and 17.

For the parallel channel with flat Au(111) walls, a large number of simulations with 120 different parameter sets $\left(h_{0},p_{n},v_{x2}^{w}\right)$ is performed. For each set, the slip velocity *v*_s_ is determined from the intersection of a linear fit to the steady-state velocity profile *v_x_*(*z*) with the wall positions. At the same time, the shear stress τ is extracted from the lateral forces on the walls. Figure 7A displays the relation between *v*_s_ and τ. The relationship *v*_s_(τ) is almost independent of the gap height *h*_0_, while there is a strong dependence on the pressure *p*_n_.

The systematic occurrence of slip in presence of a flat wall can be described with several models. The simplest model of a slip constitutive law is a linear relationship

$$\tau (v_{s})=kv_{s}$$ (9)

where *k* is the Navier friction coefficient. The inverse of *k* could serve as a measure of the slipperiness of a solid/fluid interface. However, the slip length $\lambda =v_{s}/\dot{\gamma }$ is often used to quantify slip. Under the additional assumption of a Newtonian fluid, where $\tau =\eta \dot{\gamma }$, we arrive at a relation between Navier friction and slip length λ = η/*k*. Thus, within this linear theory, the slip length λ is a constant independent of τ.

Although Eq. 9 works for certain applications, for example, in plug flow of water in carbon nanotubes (*35*) under ambient conditions, the simple linear behavior between τ and *v*_s_ does not match MD simulations of shear flow of polymers in smooth channels (*36*), and obviously, it also cannot capture the nonlinear τ(*v*_s_) relation in Fig. 7A. Consequently, more sophisticated slip laws are required.

In their pioneering work, Thompson and Troian (*37*) found a correlation between the slip length λ and the shear rate $\dot{\gamma }$ at the wall, which they expressed through a power law relationship

$$\lambda (\dot{\gamma })=\lambda_{0}{\left(1-\frac{\dot{\gamma }}{{\dot{\gamma }}_{c}}\right)}^{-0.5}$$ (10)

Here, λ_0_ and ${\dot{\gamma }}_{c}$ are constants that were used to fit the data obtained by their extensive parametric study of the shear flow in an isothermal Lennard-Jones fluid inside a channel with rigid smooth walls. Later on, Niavarani and Priezjev (*38*) showed that the Thompson-Troian slip law can be reformulated in terms of shear stress and slip velocity by considering the proportionality factor *k* = *k*(*v*_s_) to depend on slip velocity

$$\tau (v_{s})=k(v_{s})v_{s}=2\tau_{c}\left[\sqrt{1+{\left(\frac{v_{s}}{v_{c}}\right)}^{2}}-\left(\frac{\;v_{s}}{v_{c}}\right)\right]\left(\frac{\;v_{s}}{v_{c}}\right)$$ (11)

where the two coefficients $\tau_{c}=\eta {\dot{\gamma }}_{c}$ and $v_{c}=2{\dot{\gamma }}_{c}\lambda_{0}$ can be directly related to the two fitting coefficients λ_0_ and ${\dot{\gamma }}_{c}$ presented by Thompson and Troian. The characteristic stress τ_c_ represents an upper limit to the shear stress which the fluid-wall interface can sustain, while *v*_c_ simply denotes the slip velocity at which the shear stress is roughly at 80% of the maximum τ_c_.

The inverse of Eq. 11

$$v_{s}(\tau ,p)=\frac{v_{c}(p)}{2}\sqrt{\frac{\tau^{2}}{\tau_{c}(p)[\tau_{c}(p)-\tau ]}\;}$$ (12)

is a nonlinear constitutive slip law that can be fitted to the MD data in Fig. 7A. For each pressure *p*, we determine the coefficients τ_c_(*p*) and *v*_c_(*p*). Solid lines in Fig. 7A represent the individual fitted *v*_s_(τ) curves. Although some deviations can be seen for high pressures, the overall description of the underlying MD data is good. If we normalize wall shear stress τ by τ_c_ and slip velocity *v*_s_ by *v*_c_, our MD data collapse to a single master curve that follows $y=\frac{1}{2}\;\sqrt{x^{2}/(1-x)}$ with *x* = τ/τ_c_ and *y* = *v*_s_/*v*_c_ (Fig. 7B).

To arrive at a useful constitutive slip law that can be used in continuum mechanics calculation, a continuous function *v*_s_(τ, *p*) is required. The inset in Fig. 7B displays the dependencies τ_c_(*p*) and *v*_c_(*p*) on the simulated reference pressures. We arrive at a continuous *v*_s_(τ, *p*) by parametrizing τ_c_ and *v*_c_ as a function of the pressure using

$$\tau_{c}(p)=A_{\tau }p+B_{\tau }$$ (13)

and

$$v_{c}(p)=A_{v}e^{B_{v}p}$$ (14)

The parameters *A*_τ_, *B*_τ_, *A_v_*, and *B_v_* are constants that depend on the roughness of the wall and on the fluid-wall interaction. The values of these constants for hexadecane on the Au(111) surface are provided in Materials and Methods.

Instead of fitting the atomistic slip velocity *v*_s_ with the slip law Eq. 12, an Eyring type expression

$$v_{s}(\tau ,p)=v_{0}(p)\sinh\left(\frac{\tau }{\tau_{0}(p)}\right)$$ (15)

could be used (*15*, *39*–*41*). Equation 15 has the advantage of being derived from a molecular kinetic theory that considers stress assisted thermally activated forward and backward jumps of lubricant segments on solid surfaces (*15*, *39*, *40*), while Eq. 12 is only based on an empirical relationship that fits atomistic simulations of an isothermal Lennard-Jones fluid between rigid walls (*37*). Figure 7C displays fits of Eq. 15 to our parallel gold channel data. The overall agreement of the fits with the MD results is good, apart from an underestimation of the atomistic *v*_s_ for small τ and the absence of a limiting shear stress in Eq. 15, which makes the fit for high pressures difficult (see red curve in Fig. 7C). This becomes also evident in the dimensionless version of this plot—see $\frac{v_{S}}{v_{0}}$ versus $\frac{\tau }{\tau_{0}}$ relation in Fig. 7D. We describe the pressure dependence of the coefficients by

$$\tau_{0}(p)=A_{\tau_{0}}p+B_{\tau_{0}}$$ (16)

and

$$v_{0}(p)=A_{v_{0}}e^{B_{v_{0}}p+C_{v_{0}}p^{4}}$$ (17)

resulting in a satisfactory reproduction of the individual τ_0_ and *v*_0_ for the different pressures (insets in Fig. 7D). The values of the fit constants *A*_τ_0__, *B*_τ_0__, *A*_*v*_0__, *B*_*v*_0__, and *C*_*v*_0__ are provided in Materials and Methods.

Because our Au/hexadecane model is of limited practical value for real-world applications, we now consider PAO (a base oil used in engine oils) sheared between parallel walls of DLC (a coating material used in engines), because there is some indirect experimental evidence of slip in this system (*42*). We investigate 1-decene trimers (C_30_H_62_), representative of PAO4 between hydrogen-terminated amorphous carbon walls in an additional simulation campaign (see molecular setup in Fig. 1E) to check the transferability of our findings to technically relevant lubricant/material combinations. Again, a pronounced nonlinear *v*_s_ versus τ relation is observed [see *v*_s_(τ, *p*) data points in Fig. 7E] that can be fitted by both the Thompson-Troian expression (Eq. 12, see dashed curves in Fig. 7E) and the Eyring expression (Eq. 15, see solid curves in Fig. 7E). Here, the Eyring law (Eq. 15) is definitively more suitable to describe the simulations resulting in a nice collapse of the data in the dimensionless $\frac{v_{S}}{v_{0}}$ versus $\frac{\tau }{\;\tau_{0}}$ plot (Fig. 7F). As in the hexadecane/Au(111) system, the τ_0_(*p*) and *v*_0_(*p*) relations are described by Eqs. 16 and 17, respectively (see insets in Fig. 7F and fitting coefficients in Materials and Methods). Exploring the reasons for the superior fit of the Eyring model in the PAO/DLC case would exceed the scope of this article, and the underlying mechanisms should be addressed in a future investigation.

The slip laws (Eqs. 12 and 15) are introduced into the Reynolds equation (Eq. 8) via the fluid velocities at the bottom and top walls $v_{x1}=v_{x1}^{w}+v_{x1}^{s}$ and $v_{x2}=v_{x2}^{w}+v_{x2}^{s}$, respectively. The iterative algorithm to solve this equation is described in Materials and Methods. It is important to notice that we apply the slip model at both walls of the CDC only where the walls have a flat Au(111) structure. In the tilted sections of the top wall, no-slip conditions are imposed because the resulting roughness prevents any slip as shown in Fig. 6 (B and D) and discussed above.

The red solid lines in Figs. 2 and 3 represent the respective solutions *p*(*x*) and τ_top_(*x*) of the compressible Reynolds equation extended by the slip model in Eq. 12. The comparison with our atomistic CDC benchmark simulations shows that, for large gaps and small pressures, the predictive power of the RLE is approximately as good as the no-slip RLE. Conversely, the agreement between the Reynolds description and the MD for severe contact conditions strongly improves if slip is taken into account. Although there are small discrepancies of the pressure profile in the low-pressure regime at *p*_n_ = 0.2 GPa for the smallest gap height *h*_0_ = 1.4 nm, an increase in *p*_n_ leads to an excellent quantitative match between the atomistic and the slip Reynolds modeling of the CDC. The velocity profiles in Fig. 5A also exhibit overall a good agreement besides some small deviations in the central part of the channel for the most severe cases. Furthermore, the slip RLE predicts the slip velocity profiles at the top and bottom wall of the atomistic CDC (compare red lines with blue symbols Fig. 5B), confirming the accuracy of our nonlinear slip law. Results for the Eyring slip model (Eq. 15) are shown in the Supplementary Materials (figs. S13 to S18) and exhibit a similar agreement between the slip Reynolds description and the MD. This indicates a certain insensitivity in the description of the atomistic parallel channel slip velocities with different constitutive laws.

**Fig. 8.**
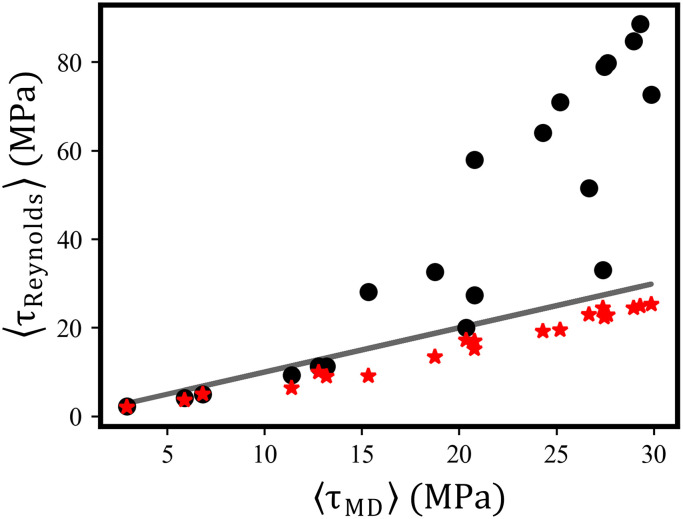
The average frictional stress at the bottom wall of the CDC. The plot compares the Reynolds results ⟨τ_Reynolds_⟩ with the atomistic ⟨τ_MD_⟩ for $v_{x1}^{w}=20\;m/s$. Black discs represent numerical calculations of the Reynolds equation with no-slip and red stars with slip boundary conditions. Note that the average frictional stress at the top wall provides an identical plot. See fig. S12 for the same plot pertaining to $v_{x1}^{w}=5\;m/s$.

Of course, it is of great importance how well our continuum models describe the overall frictional behavior of the CDC MD benchmark system. Figure 8 shows the average frictional stress at the bottom wall

$$\langle \tau_{\mathrm{Reynolds}}\rangle =\frac{1}{l_{x}}\int_{-\frac{\;l_{x}}{2}}^{\frac{l_{x}}{2}}\tau_{\mathrm{bot}}(x)dx$$ (18)

in comparison to the corresponding MD values ⟨τ_MD_⟩ for all our benchmark calculations with $v_{x1}^{w}=20\;m/s$. Clearly, the no-slip RLE severely overestimates the frictional driving forces of the atomistic CDC for large ⟨τ_MD_⟩, while the slip RLE shows only a slight underestimation.

## DISCUSSION

Two objectives were pursued in this article. First, the validity of a current state-of-the-art compressible and piezoviscous no-slip Reynolds description (*18*) of strongly loaded tribological contacts was explored. This was accomplished by comparing the lubricant shear flow in an atomistic model tribocontact (*n*-hexadecane confined by a CDC) with the results of a no-slip Reynolds equation whose equation of state and viscous law were calibrated by separate atomistic simulations of bulk representative volume elements subject to pressure or shear, respectively. It turns out that for sufficiently large minimum gaps and moderate pressures (*h*_0_ ≥ 5 nm and *p*_ref_ ≤ 0.4 GPa) the no-slip RLE provides a reasonable and robust prediction of our atomistic benchmark results. Previous MD simulations suggested that Navier-Stokes hydrodynamics can be applied down to around 10 molecular diameters (*43*) and a slight underestimation of the pressure variation along the CDC can be most likely attributed to inaccurate low-pressure Newtonian viscosities in our constitutive law.

As a second objective, we wanted to identify modifications of the no-slip Reynolds equation to extend the observed validity limits to higher pressures and smaller gaps. Various effects could be responsible for the failure of continuum predictions for extremely confined lubricants. Bulk constitutive laws can become inaccurate, because layering affects the equation of state in narrow channels (*44*), solvation pressure alters bulk viscosity laws (*45*), or viscosities are strongly increased by the emergence of solid-like states (*46*). These effects can be modeled by enhancing the equation of state (*44*) and the viscosity law (*45*, *46*) by an additional dependence on gap height.

Such extensions are not necessary for the continuum description of our CDC benchmark, as pronounced wall slip at the Au(111)/hexadecane interfaces dominates lubricant flow for *h*_0_ ≤ 5 nm. Therefore, we focused here on the question how to include wall slip properly in a Reynolds framework. A possible approach could consist of directly studying the influence of nanoscale effects on the mass flow rates with MD simulations in narrow parallel channels and introduce them via flow factors in the RLE (*47*, *48*). Such a modeling strategy would not only be able to consider wall slip, it could also catch oscillations of solvation pressure and density layering. However, it would be hard to disentangle the various mechanisms and provide a direct physical meaning of required fitting parameters.

We decided to take a more transparent route by calibrating a suitable slip constitutive law within a separate simulation campaign with hexadecane in parallel channels. By varying channel height *h*_0_, wall velocity $v_{x2}^{w}$, and normal pressure *p*_n_ in the channel, a wide range of wall slip velocities *v*_s_ and wall shear stresses τ are obtained. *v*_s_ is determined by *p*_n_ and τ, while the explicit dependence on *h*_0_ and $v_{x2}^{w}$ are negligible. This suggests that the slip velocity is simply a function of the local stress state. The local slip laws *v*_s_(τ, *p*) in Eqs. 12 and 15 describe the parallel channel data well and their introduction into a wall slip Reynolds equation greatly improves the agreement between atomistic and continuum simulations of the high-pressure flow inside our CDC geometry. Although the plug flow in the narrowest part of the *h*_0_ = 1.4 nm channel for *p*_n_ = 1 GPa is not exactly reproduced by the modified Reynolds equation, the overall agreement in the pressure profiles is surprisingly good. Obviously, slip at the Au(111)/hexadecane interface dominates the high-pressure frictional response in the CDC, and therefore, the exact description of the lubricant bulk rheology seems less important.

**Fig. 9.**
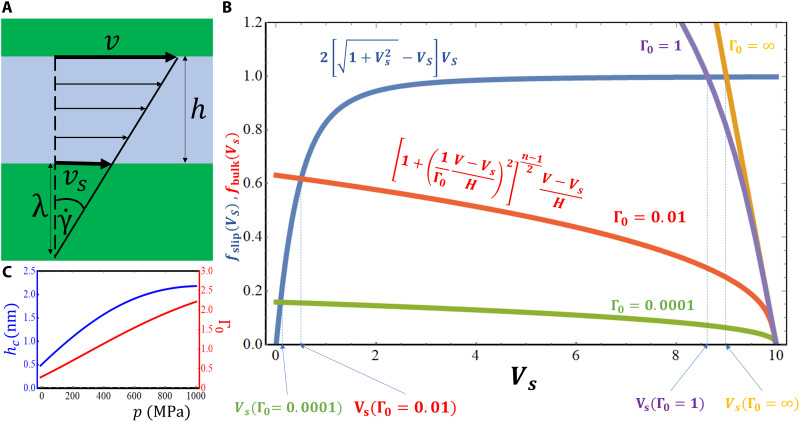
A Carreau fluid in a parallel channel with slip at the bottom wall. (**A**) Sketch of the channel along with the most important quantities. (**B**) Graphical determination of the dimensionless slip velocity *V*_s_ via intersection of the two functions *f*_slip_ (blue curve) and *f*_bulk_ for Γ_0_ = 0.0001 (green curve), Γ_0_ = 0.01 (red curve), Γ_0_ = 1 (violet curve), and Γ_0_ = ∞ (orange curve). A dimensionless top wall velocity *V* = 10, dimensionless channel height *H* = 1, and Carreau exponent *n*_Car_ = 0.4 are chosen for this example. (**C**) Pressure dependence of the critical channel height *h*_c_ and of the dimensionless Carreau shear parameter Γ_0_ for our hexadecane/Au(111) model system.

Of course, in lubricants that adhere more strongly to the walls, their bulk behavior starts to prevail, and the accuracy of constitutive viscosity laws becomes crucial. In this case, it is mandatory to consider shear thinning. To estimate when this transition from wall slip to bulk shearing occurs, we consider the shear flow in a parallel channel (Fig. 9A). We assume a shear thinning fluid obeying the Carreau constitutive law (Eq. 6) slipping at the bottom wall according to Eq. 12. The general solution of this problem is given by a Couette flow profile and force balance requires equality of the shear stresses at the bottom wall and in the fluid

$$\eta (\dot{\gamma })\dot{\gamma }=2\tau_{c}\left[\sqrt{1+{\left(\frac{v_{s}}{v_{c}}\right)}^{2}}-\left(\frac{\;v_{s}}{v_{c}}\right)\right]\left(\frac{\;v_{s}}{v_{c}}\right)\;\mathrm{with}\;\dot{\gamma }=\frac{v-v_{s}}{h}$$ (19)

After introducing $V=v/v_{c},V_{s}=v_{s}/v_{c},H=h/h_{c},{\text{Γ}}_{0}={\dot{\gamma }}_{0}/{\dot{\gamma }}_{c}$ as well as a critical shear rate ${\dot{\gamma }}_{c}=\tau_{c}/\eta_{N}$ (the shear rate for which the Newtonian shear stress in the bulk $\dot{\gamma }\eta_{N}$ equals the limiting shear stress τ_c_ at the wall) and a corresponding critical height $h_{c}=v_{c}/{\dot{\gamma }}_{c}$, we arrive at the dimensionless equation

$$\frac{V-V_{s}}{H}{\left[1+{\left(\frac{1}{{\text{Γ}}_{0}}\frac{V-V_{s}}{H}\right)}^{2}\right]}^{\frac{n_{\mathrm{car}}-1}{2}}=2V_{S}[\sqrt{1+V_{s}^{2}}-V_{S}]$$ (20)

Thus, the dimensionless wall slip velocity *V*_S_ can be deduced from the intersection point of the two functions $f_{\mathrm{slip}}(V_{s})=2V_{S}[\sqrt{1+V_{s}^{2}}-V_{S}]$ and $f_{\mathrm{bulk}}(V_{s})=\frac{V-V_{s}}{H}{\left[1+{\left(\frac{1}{{\text{Γ}}_{0}}\frac{V-V_{s}}{H}\right)}^{2}\right]}^{\frac{n_{\mathrm{car}}-1}{2}}$ (see a graphical solution of Eq. 20 for different Γ_0_ in Fig. 9B). For ${\dot{\gamma }}_{0}\to \infty$, the Carreau fluid is completely Newtonian, and thus, for large Γ_0_, shear thinning of the fluid becomes negligible. *f*_bulk_ reduces to $\frac{V-V_{s}}{H}$ for Γ_0_ → ∞, and therefore, large enough *V* (intersect of *f*_bulk_ with abscissa) and small enough *H* (inverse slope of *f*_bulk_) bring the dimensionless slip velocity *V*_s_ close to the dimensionless velocity *V* of the top wall, i.e., we obtain complete slip combined with a fluid plug sticking to the top wall. Approximately, the same behavior is still observed for Γ_0_ = 1. However, upon further decrease of Γ_0_ the dimensionless slip velocity *V*_s_ decreases substantially. In the most extreme case, Γ_0_ → 0, the slip velocity approaches zero, indicating complete sticking of the fluid at the lower wall. Complete stick can be achieved as well by sufficiently increasing *H*, because *f*_bulk_ approaches zero for *H* → ∞. Therefore, slip becomes unimportant for macroscopic channels, as expected.

It is interesting to consider the pressure dependence of the critical quantities in our hexadecane/Au(111) model (Fig. 9C). The critical channel height *h*_c _varies from 0.8 nm at *p* = 100 MPa to 2.2 nm at *p* = 1 GPa, indicating that for gap widths *h*_0_ ≫ 2 nm, no-slip boundary conditions should result in an adequate description of our model system—this is fully in agreement with our atomistic MD simulation data. Furthermore, the nondimensional Carreau shear rate parameter Γ_0_ is in the range between 0.5 and 2.2 for the pressure interval [0.1, 1] GPa. Thus, according to Fig. 9B, our hexadecane/Au(111) tribosystems are increasingly dominated by wall slip when the pressure is raised. This finding is in perfect agreement with the atomistic slip velocities shown in Fig. 5 and could explain limiting shear stresses observed in many experiments (*49*).

**Fig. 10.**
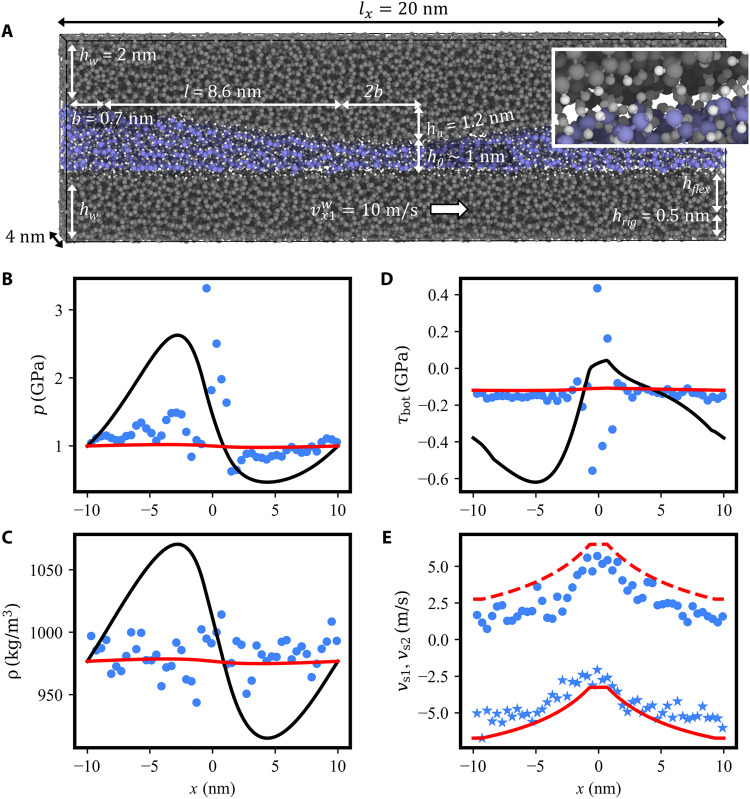
A CDC consisting of H-terminated amorphous carbon walls lubricated by 1-decene trimers. (**A**) Geometry of the simulation system, following the same color scheme as in Fig. 1E. The lateral size of the DLC CDC is scaled down by one order of magnitude compared to the gold CDC. The inset in the top right corner shows a detail of the top wall-lubricant interface, highlighting the surface roughness of the amorphous wall, which is of the order of magnitude of the C─C and C─H chemical bonds. (**B**) Pressure, (**C**) density, (**D**) frictional stress, and (**E**) slip velocities profiles of the DLC CDC evaluated by MD and RLE. Blue discs in (B) to (D) represent the data obtained from steady-state averages over MD trajectories. The results of the Reynolds equation with no-slip and slip boundary condition are displayed as black and red curves, respectively. Blue discs and stars in (E) represent the slip velocity *v*_s_(*x*) at the top and bottom wall, respectively. The results of the Reynolds equation with slip boundary conditions are displayed as solid and dashed red curves for the bottom and top wall, respectively. Large oscillations observed at the center of the CDC in the MD simulations in (B) to (D) are due to small sampling volume. See the Supplementary Materials for more details.

Although the hexadecane/gold combination represents a realistic material system, its technological relevance seems rather limited. The interface between hexadecane and Au(111) is extremely slippery. This raises the question whether such a strong wall slip can be realized in technical applications. Ewen *et al.* (*15*) observed a similar nonlinear *v*_s_(τ, *p*) law in atomistic simulations of hexadecane between hematite surfaces covered with organic friction modifiers. In addition, our additional simulations (Fig. 7C) show that the substantial wall slip of PAO in a parallel DLC channel obeys a *v*_s_(τ, *p*) law that is well described by the Eyring expression (Eq. 15). First simulations of the 1-decene trimers in a small CDC formed by H-terminated amorphous carbon walls (Fig. 10A) indicate that the RLE extended by this law can capture the flow in a technical system under boundary lubrication on a quantitative level. The pressure (Fig. 10B), density (Fig. 10C), wall frictional stress (Fig. 10D), and wall slip velocity (Fig. 10E) profiles show that the *v*_s_(τ, *p*) law of Fig. 7C is essential for an RLE description of PAO in a DLC CDC with *h*_0_ = 1 nm and *p*_n_ = 1 GPa. Note that in contrast to the hexadecane/gold system, the DLC surfaces show no orientational slip dependence, such that slip boundary conditions are used on both tilted and flat sections. Consequently, finite wall slip velocities close to those of the MD simulations are predicted by the RLE (see Fig. 10E).

This encouraging result suggests the extension of our work to more complex materials (such as DLCs, metal oxides, Si-based ceramics or surfaces covered with friction modifiers) and more complex lubricants [such as PAOs, ester oils, or water-based lubricants without or with additives (*50*)]. Such lubricant/material combinations are used in many applications and exploring the competition between wall slip, bulk shear thinning and layering effects in such systems under small gap/high pressure conditions could pave the way to a predictive modeling of friction in the boundary lubrication regime.

## MATERIALS AND METHODS

In this section, we will first present the analytical solution of the incompressible, isoviscous RLE for our CDC geometry followed by a description of the MD approach that we used to investigate the effects of compressibility, piezoviscosity, and slip at the atomistic level. Subsequently, we present the derivation of the constitutive laws and their implementation in the Reynolds solver.

### Analytical solution of the incompressible, isoviscous Reynolds equation of the CDC

In Figs. 2 and 3, an analytical RLE solution of the CDC is presented. Here, we derive this solution starting with the height function *h*(*x*) given by Eq. 1 and transform it into a dimensionless form by introducing $\xi =\frac{h_{l}}{h_{0}}$, $\kappa =\frac{l}{b}$, and $X=\frac{x}{l}$

$$H(X)=\frac{h(l)}{h_{0}}=\left\{ \begin{array}{c} 1,|X|\;<\kappa \\ 1+(\xi -1)(|X|-\;\kappa ),\kappa \leq \;|X|\;<1+\kappa \\ \xi ,1+\kappa \leq \;|X|\;\leq 1+2\kappa \end{array}\right.$$ (21)

For the stress on the top wall, we will need later

$$\frac{dh(x)}{dx}=\left\{ \begin{array}{c} 0,|x|\;<b\\ a\;\mathrm{sgn}(x),b\leq \;|x|\;<l+b\\ 0,l+b\leq \;|x| \end{array}\right.$$ (22)

where $a=\frac{h_{l}-h_{0}}{l}$. The Reynolds equation (Eq. 2) is integrated twice to yield the pressure (Eq. 3)

$$p(x)-p(0)=6\eta v_{x1}^{w}\int_{0}^{x}\left(\frac{1}{h{\left(x^{\prime }\right)}^{2}}-\frac{c}{h{\left(x^{\prime }\right)}^{3}}\right)dx^{\prime }$$

Depending on *x*, we split the integral into up to three subintegrals, e.g., for *b* ≤ *x* < *l* + *b*

$$\begin{array}{rl} & p(x)-p(0)\\ & =6\eta v_{x1}^{w}\left[\int_{0}^{b}\left(\frac{1}{{h_{0}}^{2}}-\frac{c}{{h_{0}}^{3}}\right)dx^{\prime }+\int_{b}^{x}\left(\frac{1}{h{\left(x^{\prime }\right)}^{2}}-\frac{c}{h{\left(x^{\prime }\right)}^{3}}\right)dx^{\prime }\right]\\ & =6\eta v_{x1}^{w}\left[\left(\frac{1}{{h_{0}}^{2}}-\frac{c}{{h_{0}}^{3}}\right)b+\frac{1}{a}\int_{h_{0}}^{h(x)}\left(\frac{1}{h^{\prime }2}-\frac{c}{h^{\prime }3}\right){dh}^{\prime }\right] \end{array}$$

The resulting explicit expression for the pressure

$$\begin{array}{rl} & p(x)-p(0)\\ & =6\eta v_{x1}^{w}\left\{ \begin{array}{c} \left(\frac{1}{h_{0}^{2}}-\frac{c}{h_{0}^{3}}\right)x,|x|\;<b\\ \mathrm{sgn}(x)\left\{\left(\frac{1}{h_{0}^{2}}-\frac{c}{h_{0}^{3}}\right)b+\frac{1}{2a}\left[-2\left(\frac{1}{h(x)}-\frac{1}{h_{0}}\right)+\;c\left(\frac{1}{h^{2}(x)}-\frac{1}{h_{0}^{2}}\right)\right]\right\},\\ b\leq \;|x|\;<l+b\\ \mathrm{sgn}(x)\left\{\left(\frac{1}{h_{0}^{2}}-\frac{c}{h_{0}^{3}}\right)b+\frac{1}{2a}\left[-2\left(\frac{1}{h_{l}}-\frac{1}{h_{0}}\right)+c\left(\frac{1}{h_{l}^{2}}-\frac{1}{h_{0}^{2}}\right)\right]\right\}\\ +\left(\frac{1}{h_{l}^{2}}-\frac{c}{h_{l}^{3}}\right)[x-\mathrm{sgn}(x)(l+b)],l+b\leq \;|x| \end{array}\right. \end{array}$$ (23)

can be expressed in nondimensional form

$$\begin{array}{rl} & \frac{p(x)-p(0)}{\frac{\eta v_{x1}^{w}}{h_{0}}}\frac{h_{0}}{6l}=P(X)\\ & =\left\{ \begin{array}{c} (1-C)X,|X|\;<\kappa \\ \mathrm{sgn}(X)\{(1-C)\kappa +\frac{1}{2(\xi -1)}[-2\left(\frac{1}{H}-1\right)+C\left(\frac{1}{H^{2}}-1\right)]\},\\ \kappa \;\leq \;|X|\;<1+\kappa \\ \mathrm{sgn}(X)\{(1-C)\kappa +\frac{1}{2(\xi -1)}\left[-2\left(\frac{1}{\xi }-1\right)+C\left(\frac{1}{\xi^{2}}-1\right)\right]\}\\ +\left(\frac{1}{\xi^{2}}-\frac{1}{\xi^{3}}C\right)[X-\mathrm{sgn}(X)(1+\kappa )],1+\kappa \leq \;|X| \end{array}\right. \end{array}$$ (24)

with $C=\frac{c}{h_{0}}$.

From

$$\begin{array}{rl} & p[\pm (l+2b)]-p(0)\\ & =\pm 6\eta v_{x1}^{w}\{\left(\frac{1}{h_{0}^{2}}-\frac{c}{h_{0}^{3}}\right)b+\frac{1}{2a}\left[-2\left(\frac{1}{h_{l}}-\frac{1}{h_{0}}\right)+c\left(\frac{1}{h_{l}^{2}}-\frac{1}{h_{0}^{2}}\right)\right]\\ & +\left(\frac{1}{h_{l}^{2}}-\frac{c}{h_{l}^{3}}\right)b\} \end{array}$$

and the assumption that the pressure at the entrance and at the exit of the CDC are the same [i.e., 0 = *p*(*l* + 2*b*) − *p*(−*l* − 2*b*)], a condition for *c* is calculated

$$\left(1+\frac{1}{\frac{h_{l}^{2}}{h_{0}^{2}}}\right)\frac{b}{l}-\frac{1}{\left(\frac{h_{l}}{h_{0}}-1\right)}\left(\frac{1}{\frac{h_{l}}{h_{0}}}-1\right)=\frac{c}{h_{0}}\left[\left(1+\frac{1}{\frac{h_{l}^{3}}{h_{0}^{3}}}\right)\frac{b}{l}-\frac{1}{2\left(\frac{h_{l}}{h_{0}}-1\right)}\;\left(\frac{1}{\frac{h_{l}^{2}}{h_{0}^{2}}}-1\right)\right]$$

leading to

$$\frac{c}{h_{0}}=C=\frac{(\xi^{2}+1)\kappa +\xi }{\left(\xi^{2}+\frac{1}{\xi }\right)\kappa +\frac{1+\xi }{2}}$$ (25)

#### The frictional stress on the bottom and top wall

Starting from the velocity profile that underlies the Reynolds equation

$$v_{x}(x,z)=\frac{1}{2\eta }\frac{\partial p}{\partial x}(z^{2}-hz)+\frac{v_{x1}^{w}}{h}(h-z)=v_{x1}^{w}(h-z)\left(\frac{1}{h}-\frac{1}{2\eta v_{x1}^{w}}\frac{\partial p}{\partial x}z\right)$$ (26)

inserting

$$\frac{1}{2\eta v_{x1}^{w}}\frac{dp}{dx}=\frac{3}{h^{2}}-\frac{3c}{h^{3}}$$ (27)

results in

$$v_{x}(x,z)=v_{x1}^{w}(h-z)\left[\frac{1}{h}+\left(\frac{3c}{h^{3}}-\frac{3}{h^{2}}\right)z\right]$$ (28)

as well as

$$\frac{{\partial v}_{x}(x,z)}{\partial z}=-v_{x1}^{w}\left[\frac{1}{h}+\left(\frac{3c}{h^{3}}-\frac{3}{h^{2}}\right)z\right]+v_{x1}^{w}(h-z)\left(\frac{3c}{h^{3}}-\frac{3}{h^{2}}\right)$$ (29)

and finally in

$$\tau_{\mathrm{bot}}(x)=\eta \frac{{\partial v}_{x}(x,z)}{\partial z}{|}_{z=0}=\frac{\eta v_{x1}^{w}}{h}\;\left(\frac{3c}{h}-4\right)$$ (30)

or as a dimensionless expression

$$\frac{\tau_{\mathrm{bot}}(x)}{\frac{\eta v_{x1}^{w}}{h_{0}}}=\frac{1}{H}\;\left(\frac{3C}{H}-4\right)$$ (31)

In the same way the stress on the top wall is obtained

$$\tau_{\mathrm{top}}(x)=-\eta \frac{{\partial v}_{x}(x,z)}{\partial z}{\;|}_{z=h}-p(x)\frac{dh(x)}{dx}=-\frac{\eta v_{x1}^{w}}{h}\left(2-\frac{3c}{h}\right)-p(x)\frac{dh(x)}{dx}=-\frac{\eta v_{x1}^{w}}{h_{0}}\frac{1}{H}\left(2-\frac{3C}{H}\right)-p(x)\frac{dh(x)}{dx}$$ (32)

### Molecular dynamics

#### Hexadecane in gold channels

For the MD simulations of *n*-hexadecane (C_16_H_34_) flowing in nanometer-sized gold channels, the Large-scale Atomic/Molecular Massively Parallel Simulator (LAMMPS) software suite (*51*) is used. All simulations use a velocity-Verlet algorithm with a time step of 1 fs. The embedded atom method (EAM) potential by Foiles *et al.* (*52*) models the atomic interactions within gold walls, while the Transferable Potentials for Phase Equilibria–United Atom (TraPPE-UA) force field by Martin and Siepmann (*29*) is applied for the fluid. The Lennard-Jones gold parameters from Heinz *et al.* (*53*) combined with the Lorentz-Berthelot mixing rules describe the interactions between walls and fluid [for instance resulting in an adsorption energy of hexadecane on Au(111) in agreement with the experimentally measured 16 meV per methyl group (*54*)].

Three different nanochannel geometries are considered. The first one (*A*) consists of the CDC in Fig. 1A, for which the comparison between continuum and MD is performed. The two others, used for the parameterization of the slip laws and shown in Fig. 1 (C and D), consist of parallel channels with either smooth (C) or rough surfaces (D). In all cases, the computational boxes are periodic in both the streamwise and spanwise direction. In addition to the shown setups, bulk simulations of pure *n*-hexadecane are performed for the parameterization of its density-pressure and viscosity-pressure relationships.

The geometrical parameters and conditions of the simulations are summarized in Fig. 1. For the atomistic models, the reference gap height *h*_0_ is defined as the distance between the average *z* positions of the gold layers in the top and bottom wall in contact with the fluid minus the van der Waals radius of a gold atom. In the CDC, this corresponds to the effective gap height in the narrowest section of Fig. 1A. During the equilibration and shearing steps of each simulation, the gap height is kept fixed (not taking into account small fluctuations due to elastic deformations of the gold material). Thus, the number of hexadecane molecules in the gap is initialized to ensure that the fluid pressure *p*_n_ after equilibration corresponds approximately to the desired target value.

The walls in these systems are made of crystalline gold. In the smooth case, the face-centered cubic (FCC) crystal structure is oriented to present a (111) surface in contact with the fluid. The rough surface in Fig. 1C is obtained by rotating the crystalline lattice by ϑ = 4.665° around the *y* axis, to mimic the same atomic roughness as in the slanted upper wall of the geometry in Fig. 1A. Each wall is divided into two domains. Farthest from the fluid, a rigid slab of a single atomic layer allows to apply velocity boundary conditions to the MD system, i.e., $v_{x1}^{w}=0$ at bottom wall and a finite $v_{x2}^{w}$ at the top wall. Apart from these frozen layers, the walls are thermalized at *T* = 400 K. A Langevin thermostat (*55*) with a damping time constant of 0.1 ps is used, acting only in the *z* and *y* directions to avoid affecting the system dynamics during shearing. In the fluid, viscous heat generation under shearing and limited heat removal through the walls due to wall slip (*56*, *57*) can cause thermal drift during time. To reduce this effect, which would invalidate a meaningful comparison with the steady-state and isothermal Reynolds equation, a Nosé-Hoover thermostat in the spanwise *y* direction (*58*) with a damping time constant of 0.1 ps is applied to the hexadecane molecules. With this thermostating scheme, local temperatures in the hexadecane and in the Au vary by less than 1% of the target temperature *T* = 400 K [see fig. S19 for a temperature profile in the parallel Au(111) channel with *p*_n_ = 1 GPa, *h*_0_ = 2 nm and 20 m/s wall velocity]. It should be noted that schemes that thermalize only the walls result in lubricant temperatures that substantially exceed the target temperatures of the walls (*30*, *59*)—especially for high sliding velocities. Pahlavan and Freund (*59*) had a closer look at the effect of different thermostat schemes on wall slip and concluded that an isothermal treatment of a fluid between flexible crystalline walls results in a unbounded slip length for increasing shear rates, while the temperature increase in fluids that are cooled by the walls leads to a leveling off of $\lambda (\dot{\gamma })$ for high $\dot{\gamma }$. Also in our channel with flexible Au(111) walls, the isothermal hexadecane shows an unbound asymptotic $\lambda (\dot{\gamma })$ (see fig. S20). Previously, the Thompson-Troian asymptotic behavior of $\lambda (\dot{\gamma })$ was attributed to the use of fixed walls (*60*).

#### PAO in DLC channels

A similar computational setup was adopted for the MD simulation of 1-decene trimers (C_30_H_62_) flowing between DLC surfaces. While molecular interactions are described by the original optimized potential for liquid simulations (OPLS) potential (*61*), bulk and surface atoms of the DLC walls are described using the same OPLS analytic form with parameters specifically developed to represent the geometry and the elastic constants of amorphous carbon structures (*62*). An earlier version of this force field was used to successfully model DLC walls lubricated by hydrocarbons in a previous work (*63*). The geometries of the parallel and the converging-diverging DLC channels lubricated by 1-decene trimers are shown in Figs. 1E and 10A, respectively.

The amorphous carbon walls are prepared following a melt-quench protocol (*64*). First, a random distribution of C atoms with a density of 2.5 g/cm^3^ is heated up at 9000 K for 10 ps in fully periodic orthorhombic cells. Then, the system is quenched down to 5000 K in 10 ps and then to 300 K in another 15 ps. Afterward, the system is annealed at 1000 K for 30 ps and eventually relaxed using the Fast Inertial Relaxation Engine (FIRE) algorithm (*65*), with a force tolerance of 10^−3^ eV/Å. The screened Reactive Bond Order Potential (REBO2) potential (*66*) is adopted during this protocol, as well as a time step of 0.1 fs and a Langevin thermostat with a damping time constant of 0.1 ps. The size of the simulation cells used to generate the two parallel walls is 80 Å by 40 Å by 20 Å, matching the desired size of the walls. The melt-quench protocol is performed in cells of 100 Å by 40 Å by 20 Å and 100 Å by 40 Å by 32 Å for the bottom and top walls of the DLC CDC, respectively, and these systems are replicated once along the *x* direction to obtain the desired size of the CDC. The dangling bonds of carbon atoms resulting from releasing periodicity along the vertical direction are saturated using H atoms. The surface sp^2^-hybridized C atoms bound to two H atoms are iteratively replaced by H terminations. H atoms with dangling bonds are removed. This procedure leads to a termination density of approximately 12 nm^−2^. To estimate the surface roughness of the DLC CDC walls, we calculate the RMS roughness of the bottom and the top walls by dividing the simulation cell into two-dimensional bins along the *xy* plane and using the following formula

$$R_{q}=\sqrt{\frac{1}{N_{\mathrm{bins}}}\sum_{i=1}^{N_{\mathrm{bins}}}{\left(z_{i}-\bar{z}\right)}^{2}}$$

where *N*_bins_, *z_i_*, and $\bar{z}$ represent the total number of bins, the highest *z* coordinate of the atoms in bin *i*, and the average height of the system, respectively. For this calculation, the coordinates of the top wall are flipped vertically. By choosing a size of 2 Å by 2 Å for the bins, we obtain values for *R_q_* of 0.16 and 0.43 nm for the bottom and the top walls, respectively. In particular, the first value indicates that the height variations on the flat DLC surface are of the order of the C─C bonds, while the second value represents a typical nanoscale roughness of experimental ultrasmooth DLC surfaces (*67*).

For the subsequent sliding simulations, a time step of 0.5 fs is used. The atoms belonging to the top- and bottommost domains of the parallel channel are kept rigid and fixed, respectively. A constant velocity $v_{x2}^{w}$ is applied to the rigid atoms, while the velocity of the fixed atoms $v_{x1}^{w}$ is set to zero. The thickness of these constrained regions is 5 Å. In the DLC CDC, the rigid and fixed regions are inverted, similarly to the case of the gold channels. All the systems are equilibrated for 0.5 ns at constant pressure, using the pressure-coupling algorithm described in (*68*). A constant velocity is applied to the rigid atoms, and the average height of the system is calculated during the last 250 ps of equilibration. The position of the top wall is then gradually adjusted to match the average height by imposing a constant velocity along the vertical direction for 60 ps. The sliding simulations at constant height are run for 5.0 and 2.5 ns to calculate the slip properties in the parallel channel and the CDC, respectively. The temperature of the systems is held constant by a Nosé-Hoover thermostat, with a target temperature of 330 K and a damping time constant of 0.1 ps, applied to all unconstrained atoms.

### Constitutive laws for the fluid density and viscosity as well as wall slip

#### Equation of state

We use the Tait-Murnaghan (*27*) equation of state (Eq. 5) to model the dependence of hexadecane fluid density on pressure. The coefficients ρ_0_ = 700 kg/m^3^, *p*_TM_ = 0.101 MPa, *K*_TM_ = 0.557 GPa, and *n*_TM_ = 7.33 are calibrated from equilibrium MD simulations of bulk *n*-hexadecane at *T* = 400 K (*69*, *70*).

#### Viscous constitutive law

The constitutive law for the dynamic fluid viscosity η accounts for its dependence on both the pressure and the shear rate. The overall viscosity is given by

$$\begin{array}{rl} \eta (\dot{\gamma },p) & =\eta_{0}\exp\left\{\ln\left(\frac{\eta_{0}}{\eta_{\infty }}\right)\left[-1+{\left(1+\frac{p}{p_{R}}\right)}^{z_{R}}\right]\right\}\\ & \;\times {\left[1+{\left(\frac{\dot{\gamma }}{{\dot{\gamma }}_{0}(p)}\right)}^{2}\right]}^{\frac{n_{\mathrm{Car}}(p)-1}{2}} \end{array}$$

combining the Roelands piezoviscosity formula (Eq. 29) with the Carreau equation (Eq. 28). The pressure dependence of the Carreau parameters is modeled via ${\dot{\gamma }}_{0}(p)={\dot{\gamma }}_{00}\exp(-p/p_{{\dot{\gamma }}_{0}})$ and *n*_Car_(*p*) = *n*_0_exp(−*p*/*p*_n_Car__). Here, η_∞_ = 0.06315 mPas is taken from Roelands’ original work (*71*), while the coefficients η_0_ = 0.37 mPas, *p_R_* = 0.1 GPa, *z_R_* = 0.51, ${\dot{\gamma }}_{00}$ = 13.95 GHz, $p_{{\dot{\gamma }}_{0}}=0.5022\;\mathrm{GPa},n_{0}=0.6216$, and *p*_n_Car__ = 2.028 GPa are calibrated through bulk nonequilibrium MD simulations with the SLLOD method (*72*). Note that similar viscosities are obtained by determining the slopes $\dot{\gamma }$ of the Couette velocity profiles in the constrained geometry of Fig. 1 (C and D) and applying $\eta =\tau /\dot{\gamma }$.

Parameters for PAO were obtained in a similar fashion. The compressibility, shear-thinning, and piezoviscosity of the PAO model lubricant is modeled using Eqs. 5, 6, and 7, respectively. The pressure dependence of the Carreau parameters *n*_Car_(*p*) and ${\dot{\gamma }}_{0}(p)$ is described by power law expressions $n_{\mathrm{Car}}(p)=n_{0}\;\cdot {\left(\frac{\eta_{N}(p)}{\eta_{0,n}}\right)}^{d}$and $\dot{\gamma_{0}}(p)=\dot{\gamma_{0,0}}\;\cdot {\left(\frac{\eta_{N}(p)}{\eta_{0,\dot{\gamma_{0}}}}\right)}^{c}$, where η*_N_*(*p*) is the Newtonian viscosity according to Eq. 5. The Tait-Murnaghan parameters at *T* = 330 K are given by

$$\rho_{0}=783.8\frac{\mathrm{kg}}{m^{3}},p_{\mathrm{TM}}=0.1\;\mathrm{MPa},K_{\mathrm{TM}}=1.295\;\mathrm{GPa},n_{\mathrm{TM}}=9.726$$

Furthermore, parameters for the Roelands piezoviscosity and Carreau shear-thinning equation are given by

$$\eta_{0}=0.087\;\mathrm{mPa}\cdot s,p_{R}=196.2\;\mathrm{Pa},z_{R}=0.22,\eta_{\infty }=0.06315\;\mathrm{mPa}\cdot s$$

and

$$n_{0}=0.149,\eta_{0,n}=2.004\;\mathrm{Pa}\cdot s,d=-0.196,{\dot{\gamma }}_{0,0}=25.9\mathrm{MHz},\eta_{0,{\dot{\gamma }}_{0}}=7.871\;\mathrm{Pa}\cdot s,c=-0.606$$

respectively.

#### Quantification of slip at the fluid/wall interface

To measure the slip velocity at the walls of the parallel channel, we fit a Couette profile to the mean velocity profiles after the simulation reached the steady state. The overall sampling time is 5 ns in the parallel channel hexadecane simulations. The intersections of the extrapolated Couette profile with walls provides the slip velocities $v_{x1}^{s}$ and $v_{x2}^{s}$. The position of the wall is defined where the density profile reaches zero. In the simulation with the parallel channel, the top wall is translated with a fixed velocity $v_{x2}^{w}$, and the bottom is fixed so that the overall slip velocity is the taken as the average of the absolute values of the slip velocities at both walls $v_{s}=\frac{|v_{x1}^{s}|+|v_{x2}^{s}|}{2}$. The wall shear stress is computed by measuring the overall interaction force between the fluid and the wall in *x* direction divided by the wall surface area.

#### Parameters of the slip law

For each pressure, the parameters of the *v*_s_(τ) curves in Fig. 7A are obtained by an orthogonal distance regression fit of Eq. 12 or Eq. 15 to the data points in Fig. 7A resulting in the τ_c_(*p*) and *v*_c_(*p*) data points in the inset of Fig. 7B and the τ_0_(*p*) and *v*_0_(*p*) data points in the inset of Fig. 7D, respectively. In a second step, these data points are fitted by Eqs. 13 and 14 or Eqs. 16 and 17. This yields the following parameters

$$A_{\tau }=8.058\times 10^{-3},B_{\tau }=17.44\;\mathrm{MPa},A_{v}=24.126\;m/s,\mathrm{and}\;B_{v}=-2.555\;\mathrm{GP}a^{-1}$$

and

$$\begin{array}{c} A_{\tau_{0}}=-5.291\times 10^{-3},B_{\tau_{0}}=7.47\;\mathrm{MPa}\\ A_{v_{0}}=6.038\;m/s,B_{v_{0}}=-4.656\;{\mathrm{GPa}}^{-1},\mathrm{and}\;C_{v_{0}}=-5.881\;{\mathrm{GPa}}^{-4} \end{array}$$

The slip in the DLC/PAO system was described by the Eyring expression (Eq. 15) and the relations τ_0_(*p*) and *v*_0_(*p*) are described by Eqs. 16 and 17 with parameters

$$A_{\tau_{0}}=4.088\times 10^{-3},B_{\tau_{0}}=9.66\;\mathrm{MPa}$$

$$A_{v_{0}}=0.072\;m/s,B_{v_{0}}=-1.094\;{\mathrm{GPa}}^{-1},\mathrm{and}\;C_{v_{0}}=-2.382\;{\mathrm{GPa}}^{-4}$$

### Numerical solution of the Reynolds equation

The Reynolds equation (Eq. 8) is coupled with the equation of state (Eq. 5), the viscous constitutive law (Eqs. 6 and 7), and the wall slip laws (Eq. 12 or Eq. 15). The system is discretized through a finite volume approach according to Arghir *et al.* (*73*) and solved with an iterative approach with Anderson relaxation (*74*) for the pressure profile *p*(*x*) and simple relaxation for the viscosities and slip velocities. For a given gap height *h*(*x*), and after the initialization of the viscosity, density, and slip velocity profiles, the solution of the algorithm follows this scheme:

1) Solution of the compressible Reynolds equation (Eq. 8).

2) Computation of the density and viscosity.

3) Computation of the shear rate at the bottom and top wall.

4) Computation of the shear stress at both walls $\tau =\eta \dot{\gamma }$

5) Application of the slip constitutive law

$$v_{s}(\tau ,p)=\frac{v_{c}(p)}{2}\sqrt{\frac{\tau^{2}}{\tau_{c}(p)[\tau_{c}(p)-\tau ]}\;}\;\mathrm{or}\;v_{s}(\tau ,p)=v_{0}(p)\sinh\left(\frac{\tau }{\tau_{0}(p)}\right)$$

where the coefficients τ_c_ and *v*_c_ (or τ_0_ and *v*_0_) have been updated according to the local pressure in each domain point. After this step, the slip velocities $v_{x1}^{s}(x)$ and $v_{x2}^{s}(x)$ at the bottom and top wall are available.

1) Anderson relaxation of the pressure profile and simple relaxation of the local viscosity and slip velocities.

2) Computation of the pressure residuum

$$\varepsilon_{p}=\frac{\int_{-\frac{l_{x}}{2}}^{\frac{l_{x}}{2}}dx|p^{\mathrm{new}}(x)-p^{\mathrm{old}}(x)|}{\int_{-\frac{l_{x}}{2}}^{\frac{l_{x}}{2}}dx|p^{\mathrm{new}}(x)|}$$

3) If the residuum ɛ*_p_* is bigger than the tolerance, go back to point 1.

The continuum simulations in this work used a 250-point discretization grid, an Anderson memory of five previous pressures and an Anderson α = 0.1. An underrelaxation factor of 0.01 for the viscosity and slip velocities is used. Reaching convergence at residuum ɛ*_p_* < 10^−7^, typically require 200 to 2000 iteration steps and approximately 5 to 50 s on a single core of a personal laptop.
