# Supplementary material for: Toward a continuum description of lubrication in highly pressurized nanometer-wide constrictions: The importance of accurate slip laws
Source: Sci Adv. 2023 Dec 1;9(48):eadi2649. doi: 10.1126/sciadv.adi2649 (PMC10691767; doi:10.1126/sciadv.adi2649)
Supplement: Supplementary file 1 — Figs. S1 to S20 [file sciadv.adi2649_sm.pdf]

Science Advances
AAAS

Supplementary Materials for

Toward a continuum description of lubrication in highly pressurized nanometer-wide constrictions: The importance of accurate slip laws

Andrea Codrignani *et al.*

Corresponding author: Kerstin Falk, kerstin.falk@iwm.fraunhofer.de;
Michael Moseler, michael.moseler@iwm.fraunhofer.de

Sci. Adv. **9**, eadi2649 (2023)
DOI: 10.1126/sciadv.adi2649

This PDF file includes:

Figs. S1 to S20

Supplementary Material

In this document we show the following supplementary information to the paper:

- Fig. S1: Pressure profiles in the CDC for $\boldsymbol{v_{x1}^{w}} = \mathbf{5}$ m/s.
- Fig. S2: Density profiles in the CDC for $\boldsymbol{v_{x1}^{w}} = \mathbf{20}$ m/s.
- Fig. S3: Density profiles in the CDC for $\boldsymbol{v_{x1}^{w}} = \mathbf{5}$ m/s.
- Fig. S4: Shear stress profiles on top wall of the CDC for $\boldsymbol{v_{x1}^{w}} = \mathbf{20}$ m/s.
- Fig. S5: Shear stress profiles on top wall of the CDC for $\boldsymbol{v_{x1}^{w}} = \mathbf{5}$ m/s.
- Fig. S6: Shear stress profiles on bottom wall of the CDC for $\boldsymbol{v_{x1}^{w}} = \mathbf{20}$ m/s.
- Fig. S7: Shear stress profiles on bottom wall of the CDC for $\boldsymbol{v_{x1}^{w}} = \mathbf{5}$ m/s.
- Fig. S8: Velocity profiles in the CDC for $\boldsymbol{v_{x1}^{w}} = \mathbf{20}$ m/s.
- Fig. S9: Velocity profiles in the CDC for $\boldsymbol{v_{x1}^{w}} = \mathbf{5}$ m/s.
- Fig. S10: Slip velocity profiles at top and bottom wall in the CDC for $\boldsymbol{v_{x1}^{w}} = \mathbf{20}$ m/s.
- Fig. S11: Slip velocity profiles at top and bottom wall in the CDC for $\boldsymbol{v_{x1}^{w}} = \mathbf{5}$ m/s.
- Fig. S12: The average shear stress at the bottom wall of the CDC for $\boldsymbol{v_{x1}^{w}} = \mathbf{5\ m/s}$.
- Fig. S13. Pressure profiles in the gold/hexadecane CDC for $\boldsymbol{v_{x1}^{w}} = \mathbf{5}$ m/s and two different slip laws.
- Fig. S14. Pressure profiles in the gold/hexadecane CDC for $\boldsymbol{v_{x1}^{w}} = \mathbf{20}$ m/**s** and two different slip laws.
- Fig. S15. Frictional stress profiles at bottom wall of the gold/hexadecane CDC for $\boldsymbol{v_{x1}^{w}} = \mathbf{5}$ m/s and two different slip laws.
- Fig. S16. Frictional stress profiles at bottom wall of the gold/hexadecane CDC for $\boldsymbol{v_{x1}^{w}} = \mathbf{20}$ m/s and two different slip laws.
- Fig. S17. Slip velocity profiles at bottom and top wall of the gold/hexadecane CDC for $\boldsymbol{v_{x1}^{w}} = \mathbf{5}$ m/s and two different slip laws.

- Fig. S18. Slip velocity profiles at bottom and top wall of the gold/hexadecane CDC for $\boldsymbol{v_{x1}^{w}} = \mathbf{20}$ m/s and two different slip laws.

- Fig. S19. Temperature profile in the parallel Au(111) channel with $\boldsymbol{p_n} = \mathbf{1}$ GPa and $\boldsymbol{h_0} = \mathbf{2}$ nm and 20 m/s wall velocity.

- Fig. S20. Slip length versus shear rate in the hexadecane confined by the Au(111) channel with parallel walls.

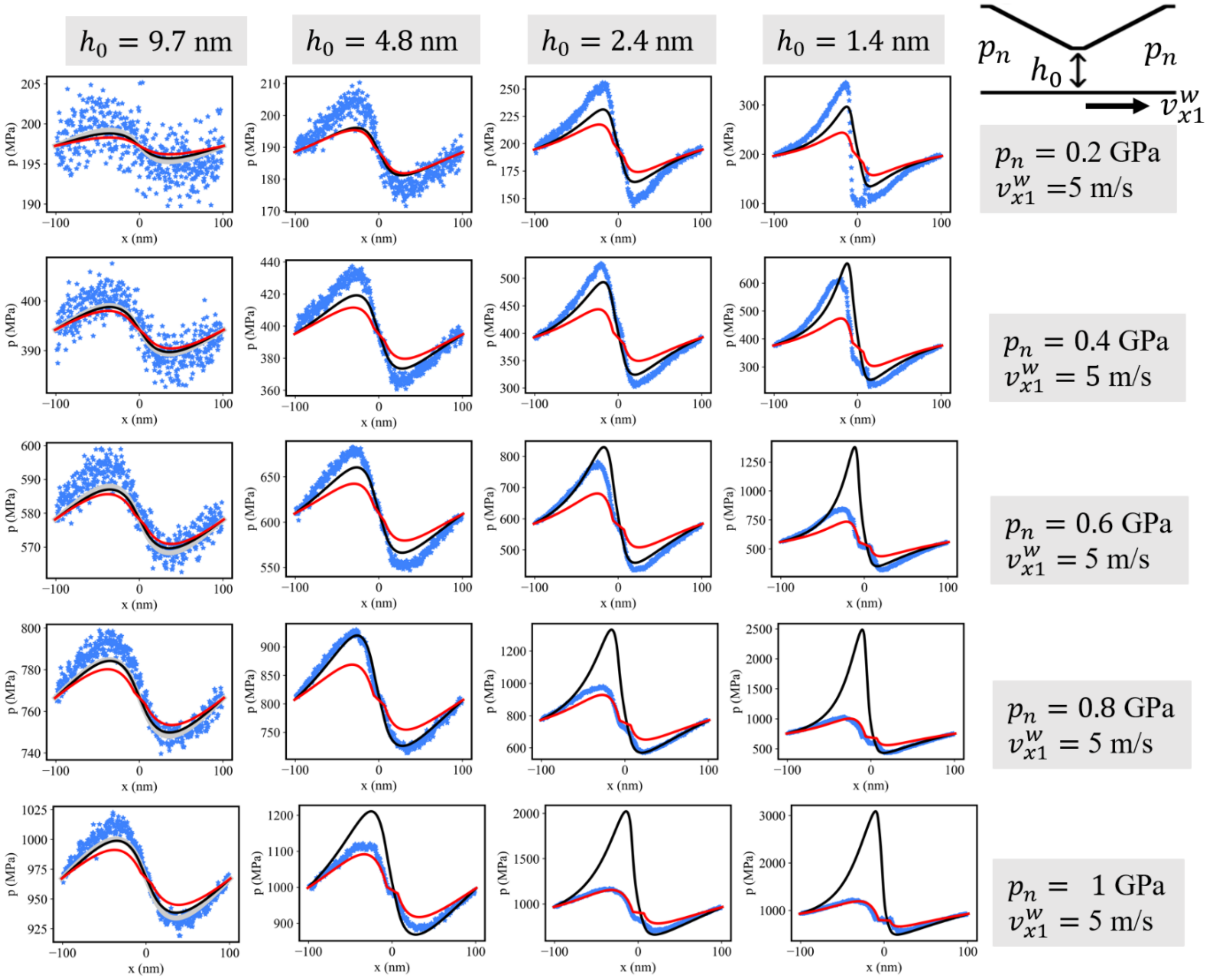

Fig. S1. Pressure profile in the gold/hexadecane CDC for $\boldsymbol{v_{x1}^{w}} = \mathbf{5}$ m/s. $p(x)$ is shown for minimum gap heights $h_0 = [9.7, 4.8, 2.4, 1.4]$ nm and nominal normal pressures $p_n = [0.2, 0.4, 0.6, 0.8, 1.0]$ GPa. Blue stars represent the pressure $p(x)$ obtained from 10 ns steady state averages over molecular dynamics trajectories. Grey curves in the first column are the exact analytic solution for the incompressible, isoviscous Reynolds equation. The numerical solutions of the Reynolds equation with no-slip and slip boundary condition are displayed as black and red curves, respectively.

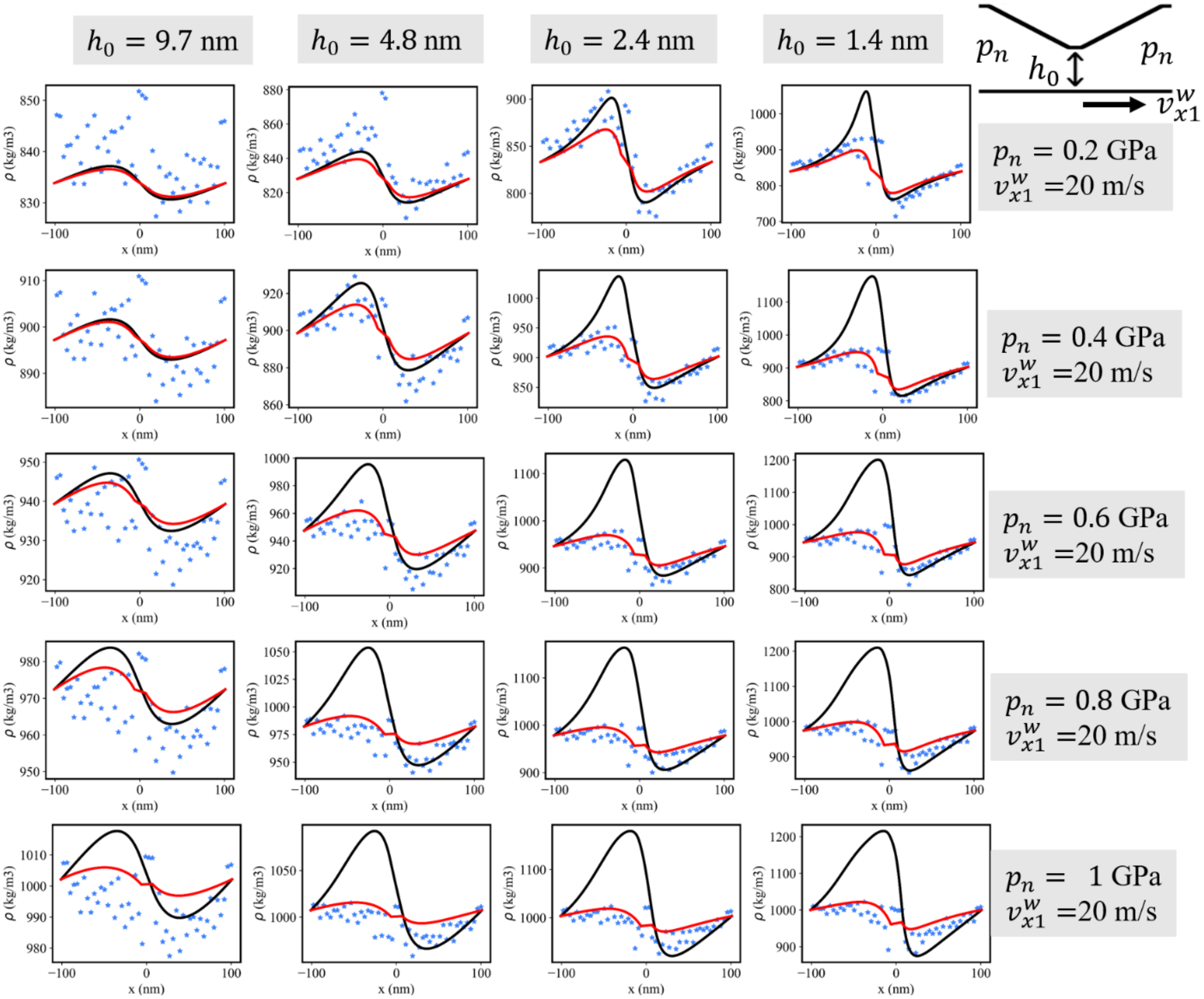

Fig. S2. Density profiles in the gold/hexadecane CDC for $v_{x1}^w = 20$ m/s. $\rho(x)$ is shown for minimum gap heights $h_0 = [9.7, 4.8, 2.4, 1.4]$ nm and nominal normal pressures $p_{\mathrm{n}} = [\ 0.2, 0.4, 0.6, 0.8, 1.0]$ GPa. Blue stars represent the result obtained from 10 ns steady state averages over molecular dynamics trajectories. The numerical solutions of the Reynolds equation with no-slip and slip boundary condition are displayed as black and red curves, respectively.

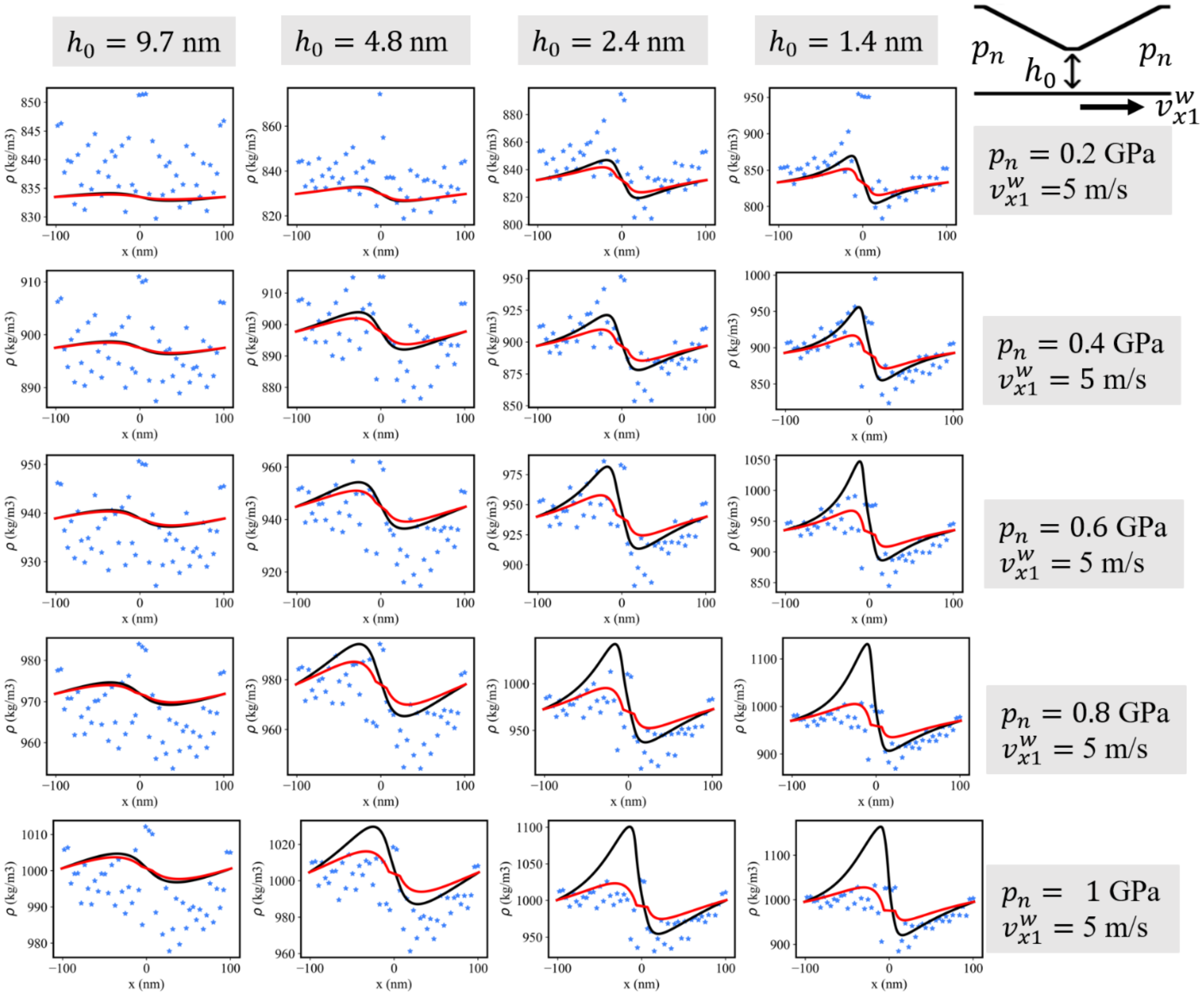

Fig. S3. Density profiles in the gold/hexadecane CDC for $v_{x1}^w = 5$ m/s. $\rho(x)$ is shown for minimum gap heights $h_0 = [9.7, 4.8, 2.4, 1.4]$ nm and nominal normal pressures $p_\mathrm{n} = [0.2, 0.4, 0.6, 0.8, 1.0]$ GPa. Blue stars represent the result obtained from 10 ns steady state averages over molecular dynamics trajectories. The numerical solutions of the Reynolds equation with no-slip and slip boundary condition are displayed as black and red curves, respectively.

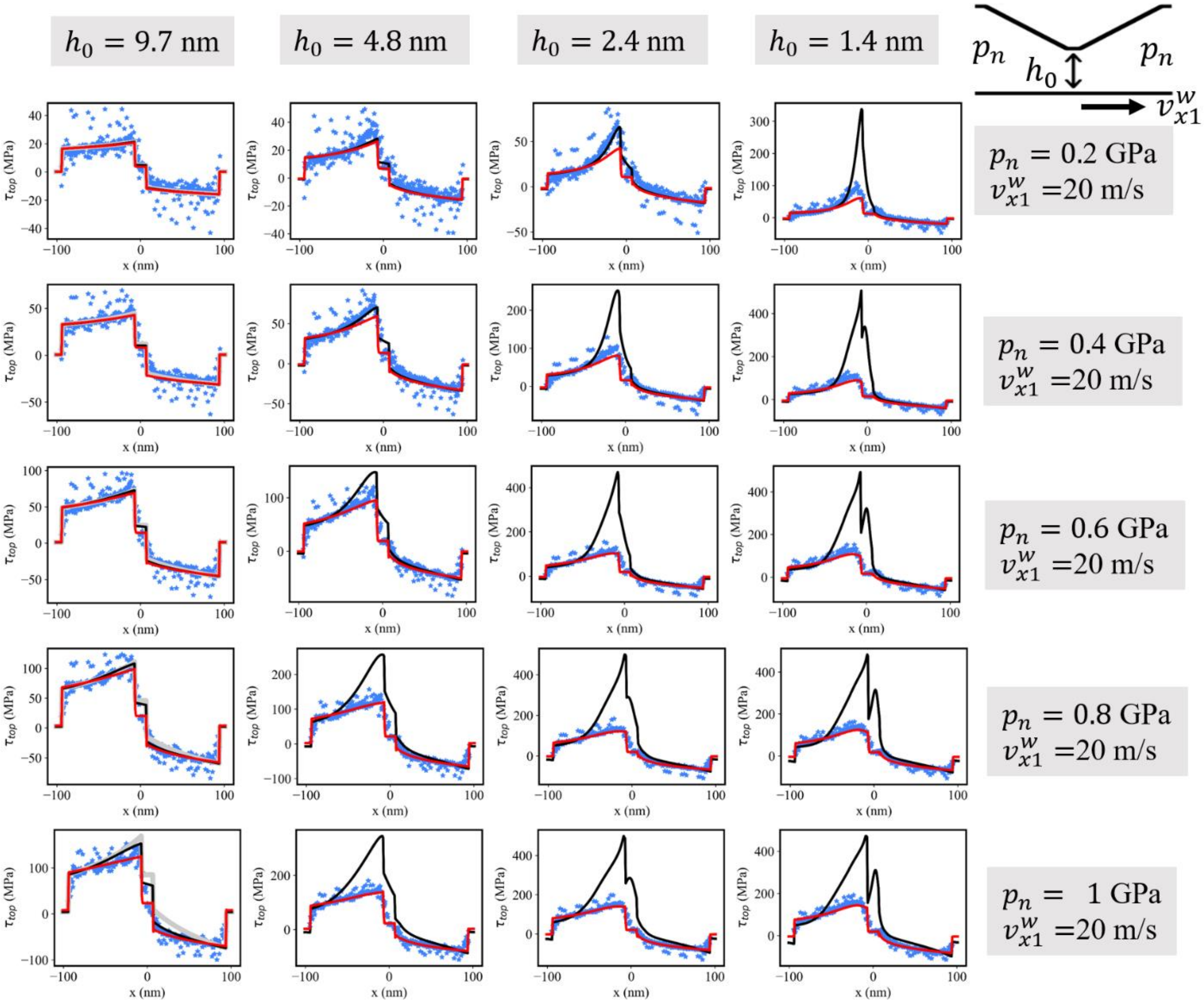

Fig. S4. Frictional stress profiles on top wall of the gold/hexadecane CDC for $v_{x1}^w = 20$ m/s. $\tau_{top}(x)$ is shown for minimum gap heights $h_0 = [9.7, 4.8, 2.4, 1.4]$ nm and nominal normal pressures $p_\text{n} = [\,0.2, 0.4, 0.6, 0.8, 1.0]$ GPa. Blue stars represent results obtained from 10 ns steady state averages over molecular dynamics trajectories. Grey curves in the first column are the exact analytic solution for the incompressible, isoviscous Reynolds equation. The numerical solutions of the Reynolds equation with no-slip and slip boundary condition are displayed as black and red curves, respectively.

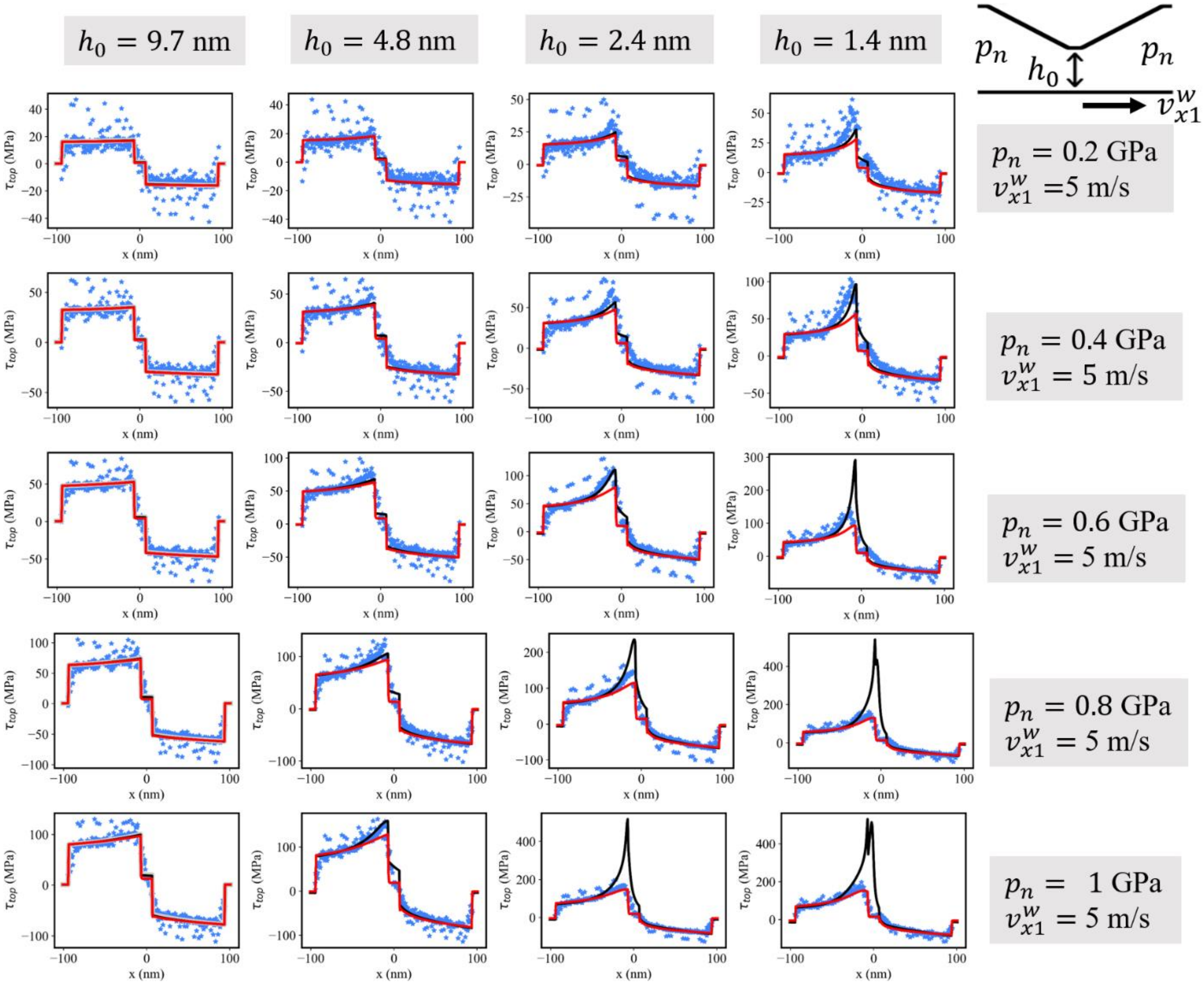

Fig. S5. Frictional stress profiles on top wall in the gold/hexadecane CDC for $v_{x1}^w = 5$ m/s. $\tau_{top}(x)$ is shown for minimum gap heights $h_0 = [9.7, 4.8, 2.4, 1.4]$ nm and nominal normal pressures $p_\mathrm{n} = [0.2, 0.4, 0.6, 0.8, 1.0]$ GPa. Blue stars represent results obtained from 10 ns steady state averages over molecular dynamics trajectories. Grey curves in the first column are the exact analytic solution for the incompressible, isoviscous Reynolds equation. The numerical solutions of the Reynolds equation with no-slip and slip boundary condition are displayed as black and red curves, respectively.

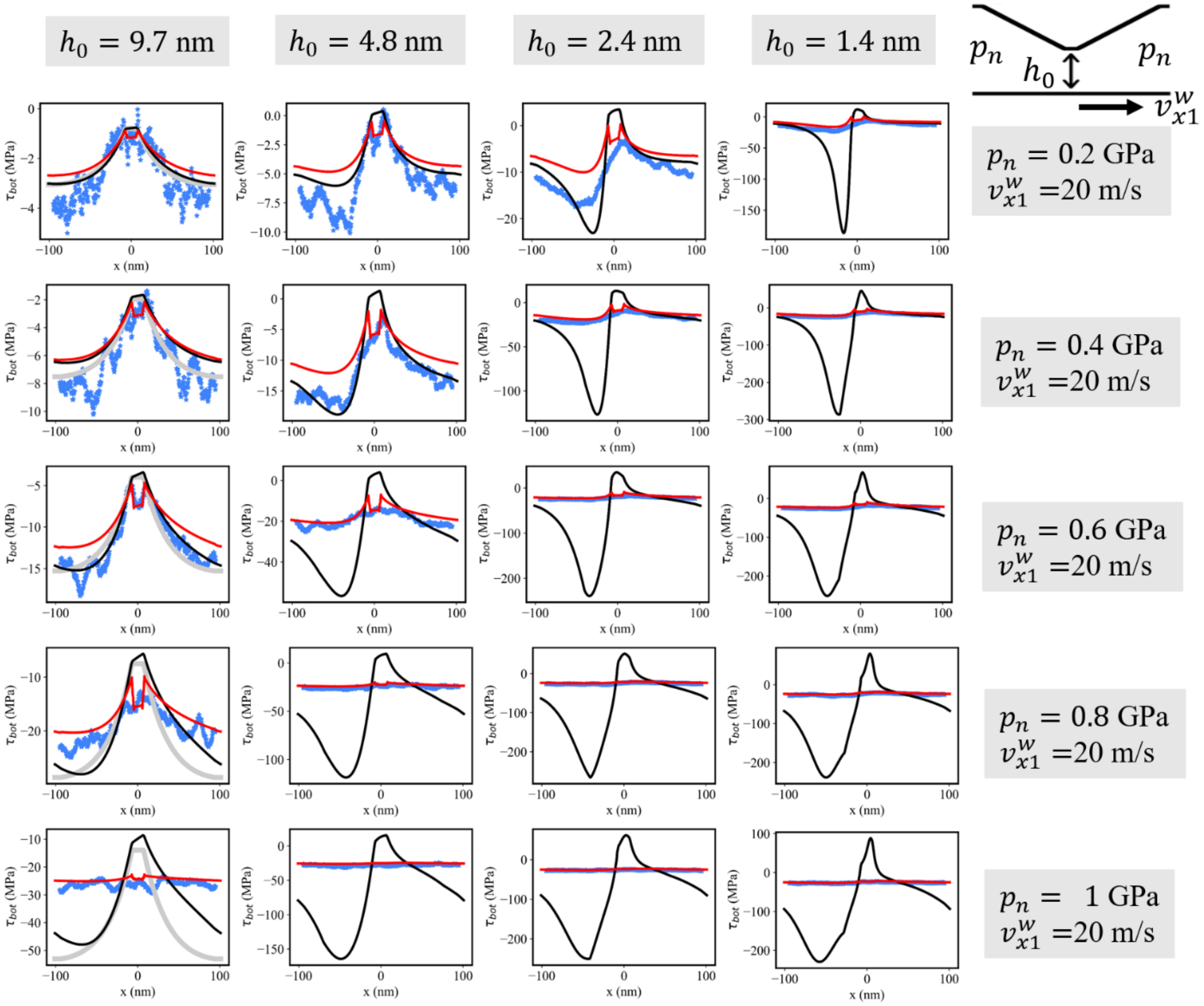

Fig. S6. Frictional stress profiles on bottom wall of the gold/hexadecane CDC gold channel for $v_{x1}^w = 20$ m/s. $\tau_{bot}(x)$ is shown for minimum gap heights $h_0 = [9.7, 4.8, 2.4, 1.4]$ nm and nominal normal pressures $p_\mathrm{n} = [0.2, 0.4, 0.6, 0.8, 1.0]$ GPa. Blue stars represent results obtained from 10 ns steady state averages over molecular dynamics trajectories. Grey curves in the first column are the exact analytic solution for the incompressible, isoviscous Reynolds equation. The numerical solutions of the Reynolds equation with no-slip and slip boundary condition are displayed as black and red curves, respectively.

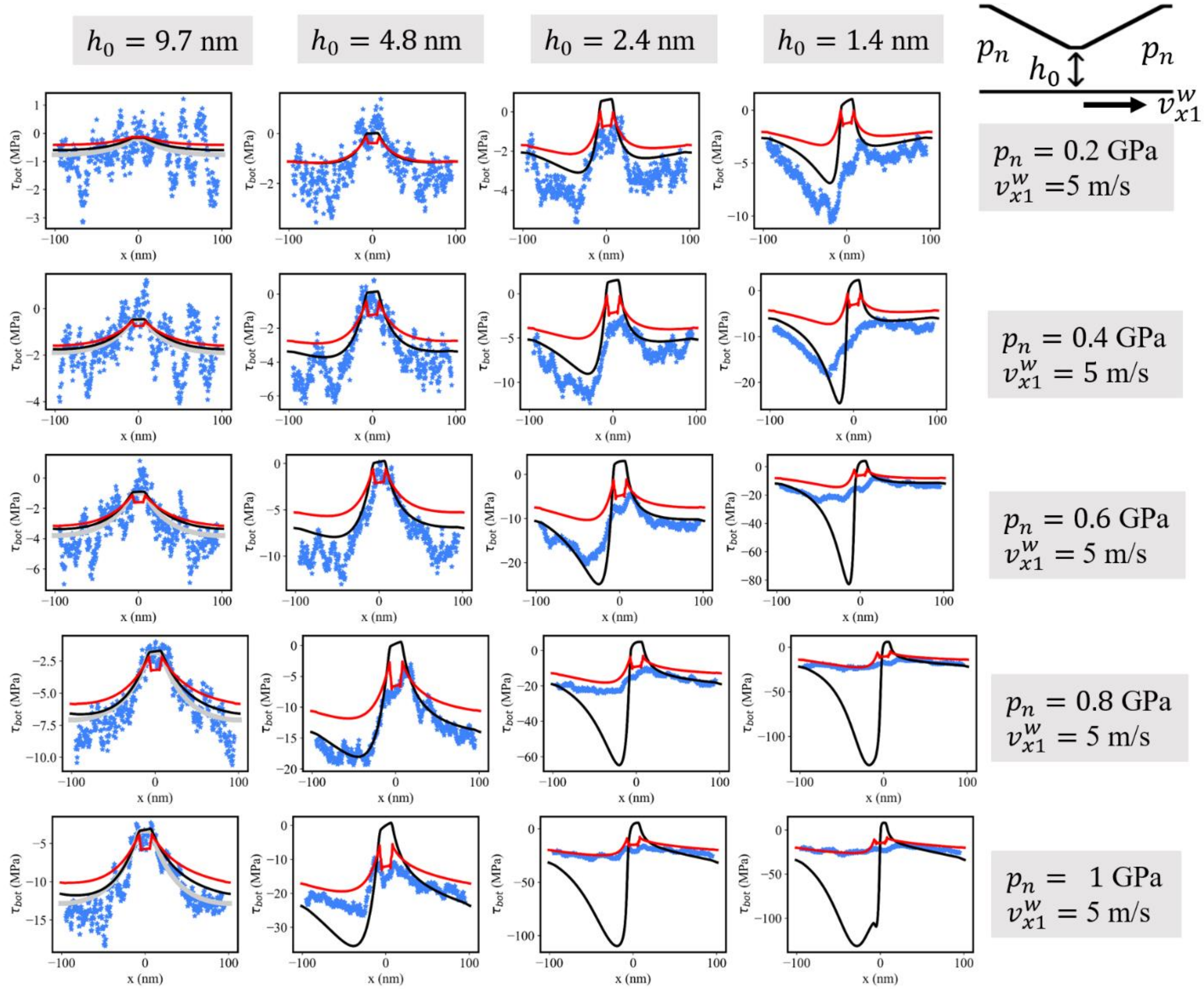

Fig. S7. Frictional stress profiles on bottom wall of the gold/hexadecane CDC for $v_{x1}^w = 5$ m/s. $\tau_{bot}(x)$ is shown for minimum gap heights $h_0 = [9.7, 4.8, 2.4, 1.4]$ nm and nominal normal pressures $p_\mathrm{n} = [0.2, 0.4, 0.6, 0.8, 1.0]$ GPa. Blue stars represent results obtained from 10 ns steady state averages over molecular dynamics trajectories. Grey curves in the first column are the exact analytic solution for the incompressible, isoviscous Reynolds equation. The numerical solutions of the Reynolds equation with no-slip and slip boundary condition are displayed as black and red curves, respectively.

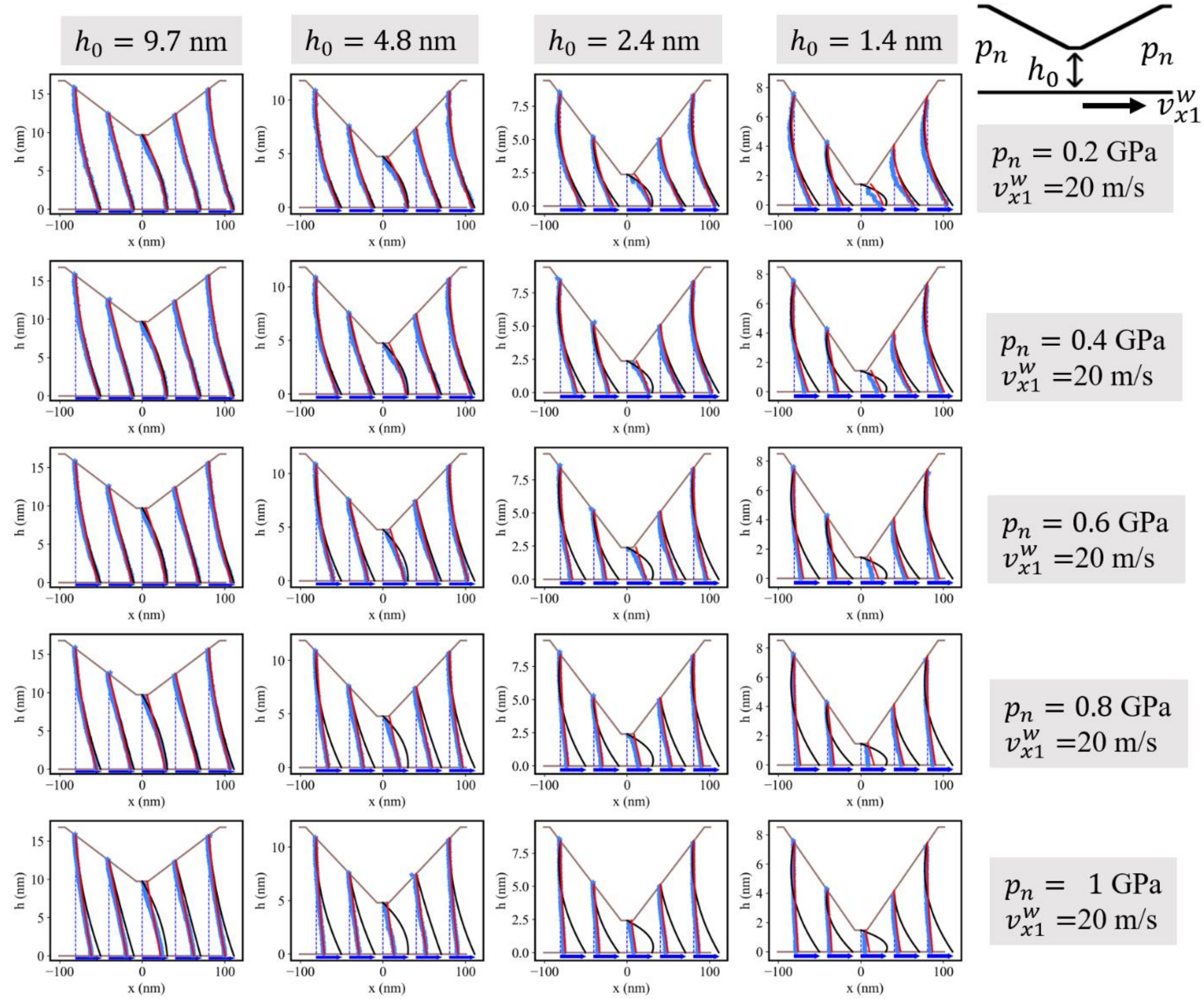

Fig. S8. Velocity profiles in the gold/hexadecane CDC for $v_{x1}^{w} = 20$ m/s. A velocity of the lower gold wall of $v_{x1}^{w} = 20$ m/s (depicted as blue arrows), minimum gap heights $h_0 = [9.7, 4.8, 2.4, 1.4]$ nm and normal pressures $p_\mathrm{n} = [0.2, 0.4, 0.6, 0.8, 1.0]$ GPa are shown. Blue dots represent the velocities $v_x(x, z)$ at 5 different x-positions obtained from 10 ns steady state averages over molecular dynamics trajectories. Results of the Reynolds equation with no-slip and slip boundary condition are displayed as black and red curves, respectively.

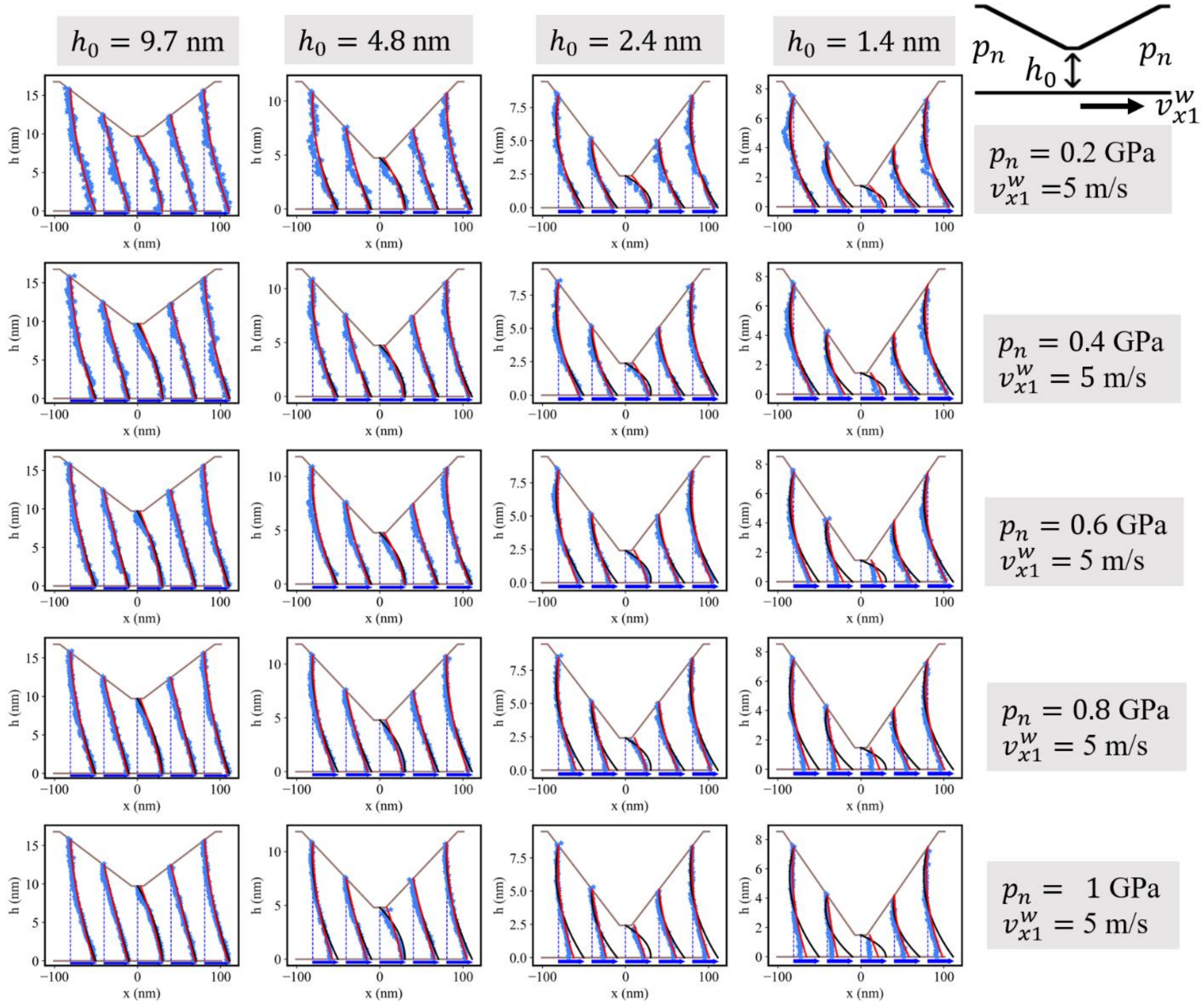

Fig. S9. Velocity profiles in the gold/hexadecane CDC for $v_{x1}^w = 5$ m/s. A velocity of the lower gold wall of $v_{x1}^w = 5$ m/s (depicted as blue arrows), minimum gap heights $h_0 = [9.7, 4.8, 2.4, 1.4]$ nm and normal pressures $p_\mathrm{n} = [0.2, 0.4, 0.6, 0.8, 1.0]$ GPa are shown. Blue dots represent the velocities $v_x(x, z)$ at 5 different x-positions obtained from 10 ns steady state averages over molecular dynamics trajectories. Results of the Reynolds equation with no-slip and slip boundary condition are displayed as black and red curves, respectively.

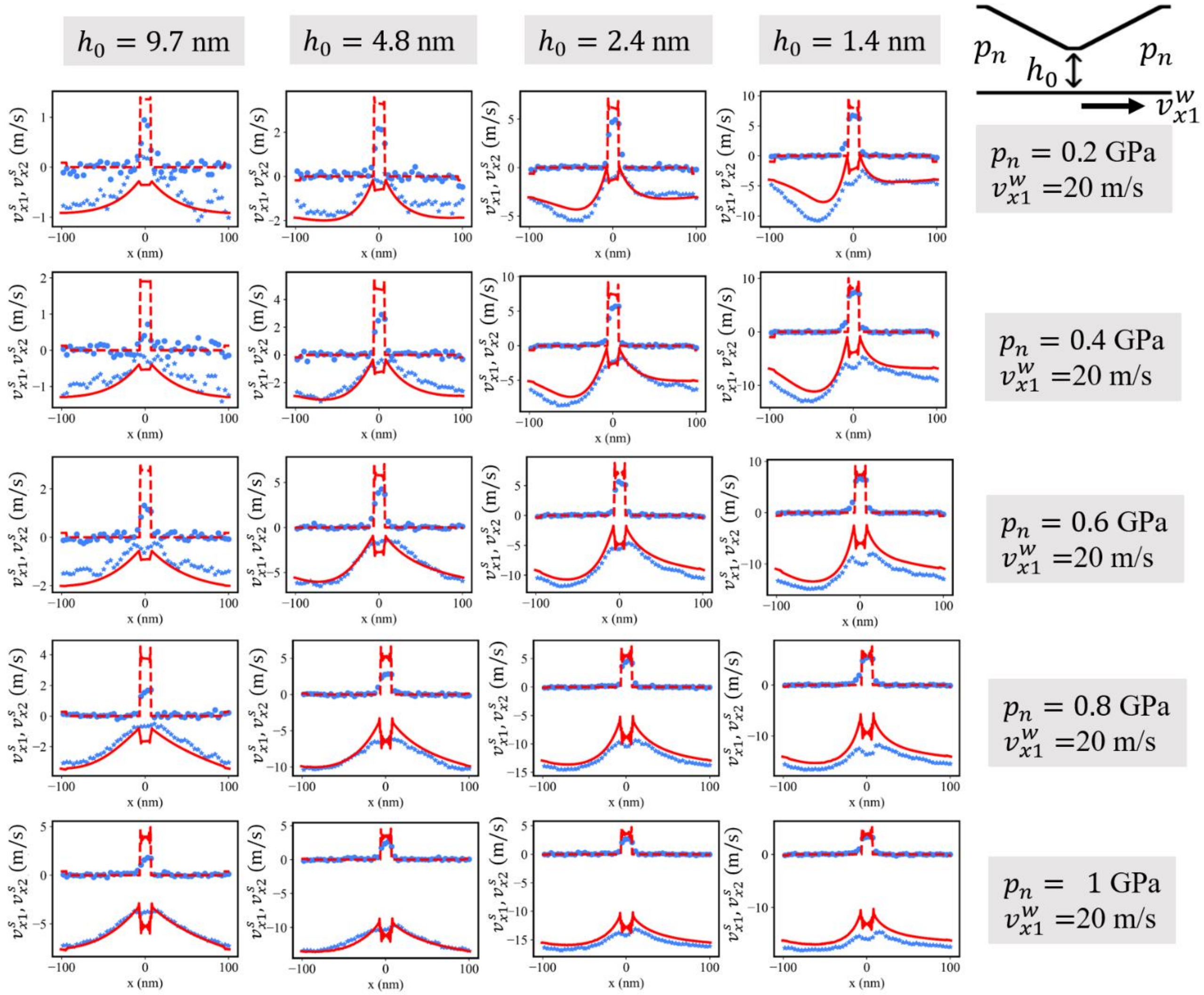

Fig. S10. Slip velocity profiles at top and bottom wall in the gold/hexadecane CDC for $v_{x1}^w = 20$ m/s. $v_{x1}^s(x)$ and v_{x2}^s are shown for minimum gap heights $h_0 = [9.7, 4.8, 2.4, 1.4]$ nm and nominal normal pressures $p_\mathrm{n} = [0.2, 0.4, 0.6, 0.8, 1.0]$ GPa. Blue stars represent results obtained from 10 ns steady state averages over molecular dynamics trajectories. The numerical solutions of the Reynolds equation with no-slip and slip boundary condition are displayed as black and red curves, respectively.

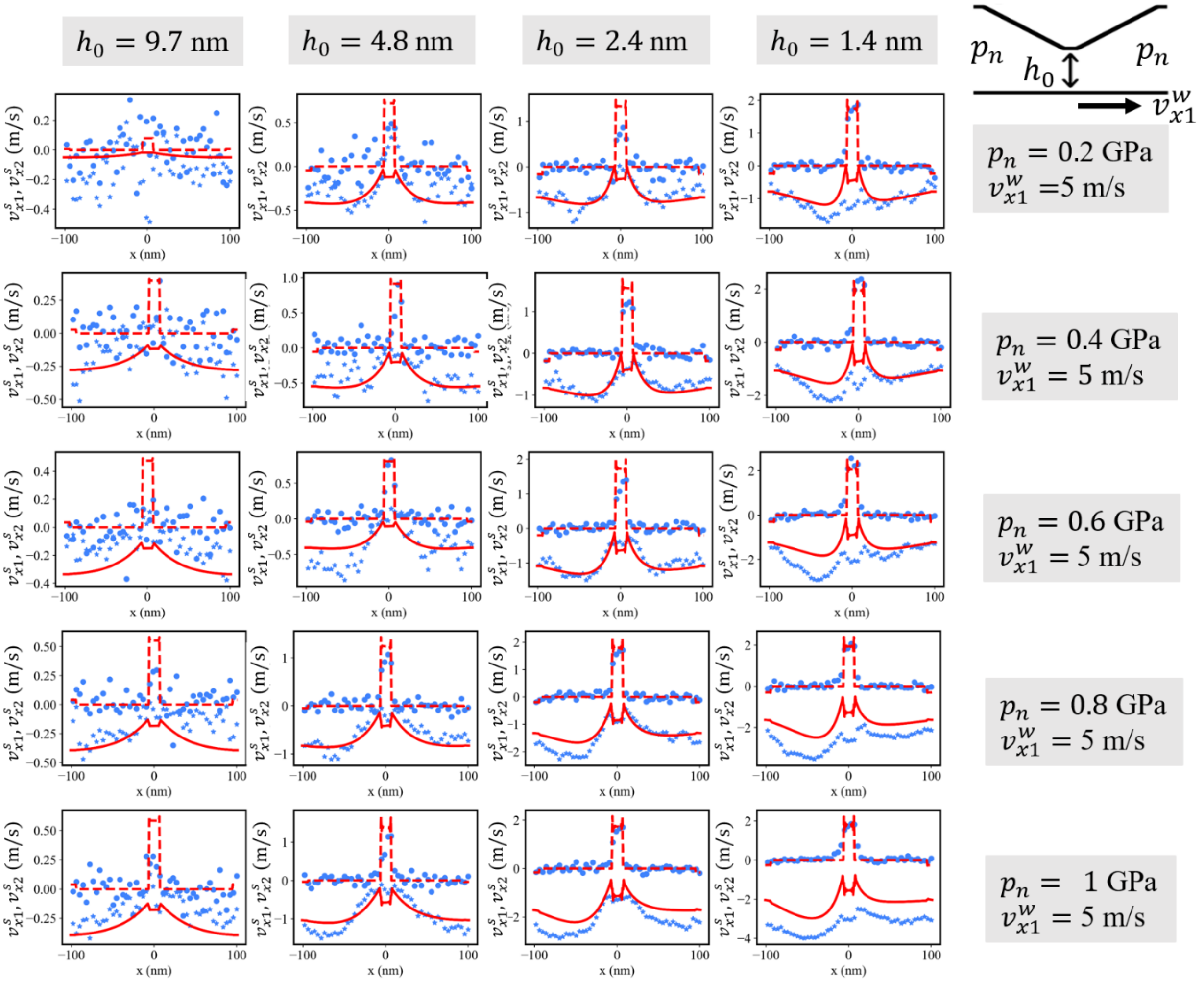

Fig. S11. Slip velocity profiles at top and bottom wall in the gold/hexadecane CDC for v^w_{x1} = 5 m/s. $v^s_{x1}(x)$ and v^s_{x2} are shown for minimum gap heights $h_0 = [9.7, 4.8, 2.4, 1.4]$ nm and nominal normal pressures $p_n = [0.2, 0.4, 0.6, 0.8, 1.0]$ GPa. Blue stars represent results obtained from 10 ns steady state averages over molecular dynamics trajectories. The numerical solutions of the Reynolds equation with no-slip and slip boundary condition are displayed as black and red curves, respectively.

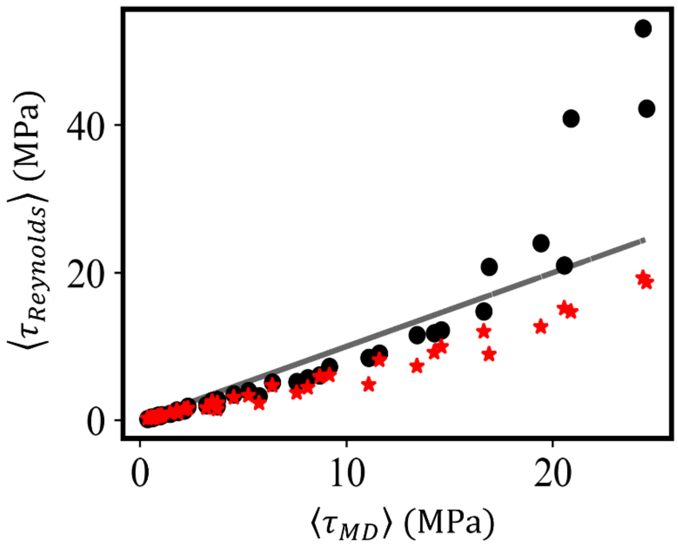

Fig. S12. The average frictional stress at the bottom wall of the gold/hexadecane CDC for $\boldsymbol{v_{x1}^{w} = 5}$ m/s. The plot compares the Reynolds results $\langle \tau_{Reynolds} \rangle$ with the atomistic $\langle \tau_{MD} \rangle$. Black discs represent numerical calculations of the Reynolds equation with no-slip and red stars with slip boundary conditions. Note, that the average shear stress at the top wall provide an identical plot.

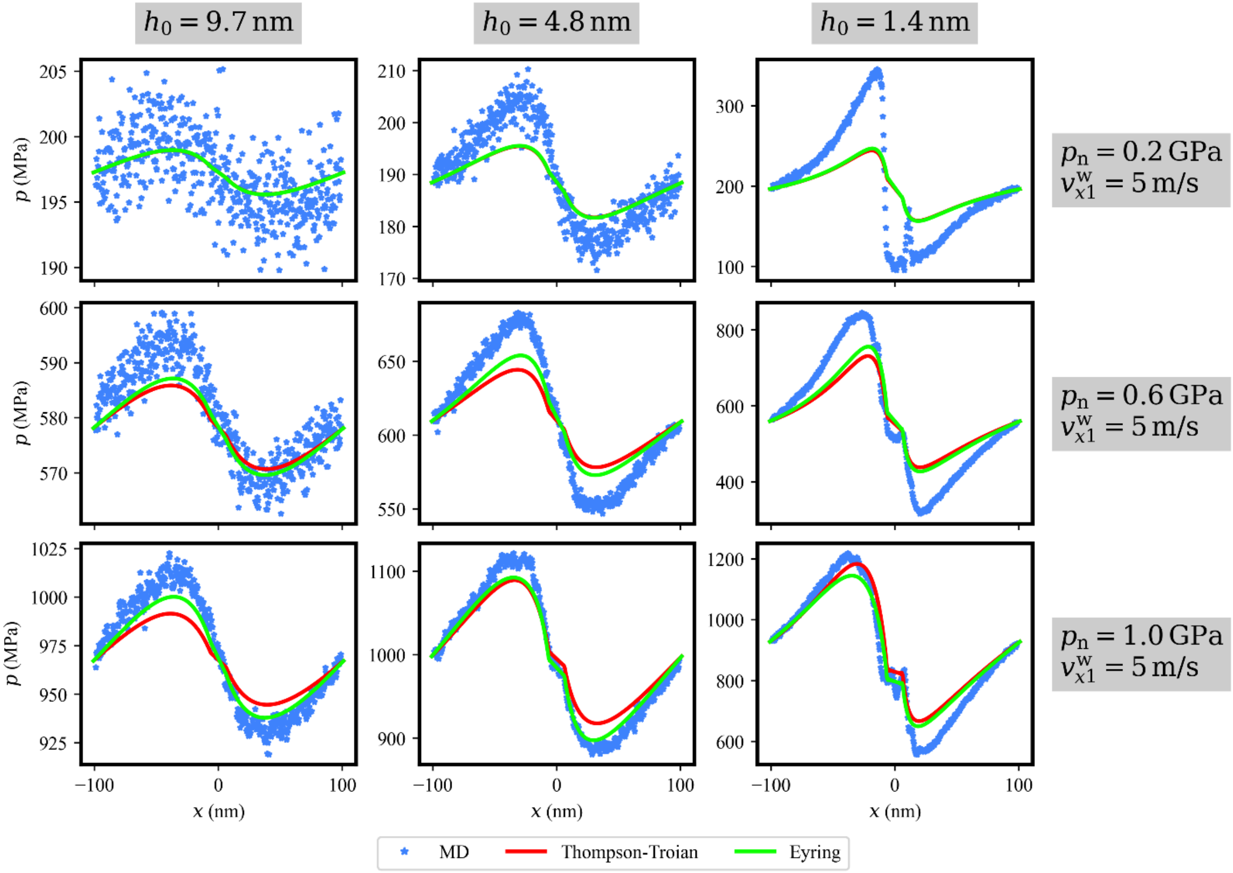

Fig. S13. Pressure profiles in the gold/hexadecane CDC for $v_{x1}^{w} = 5$ m/s and two different slip laws. $p(x)$ is shown for minimum gap heights $h_0 = [9.7, 4.8,\ 1.4]$ nm and nominal normal pressures $p_\mathrm{n} = [0.2,\ 0.6, 1.0]$ GPa. Blue stars represent the pressure $p(x)$ obtained from 10 ns steady state averages over molecular dynamics trajectories. Red curves are the same as in Fig. S1 for the Thompson-Troian slip law and green curves correspond to the numerical solution of the Reynolds equation with Eyring slip law.

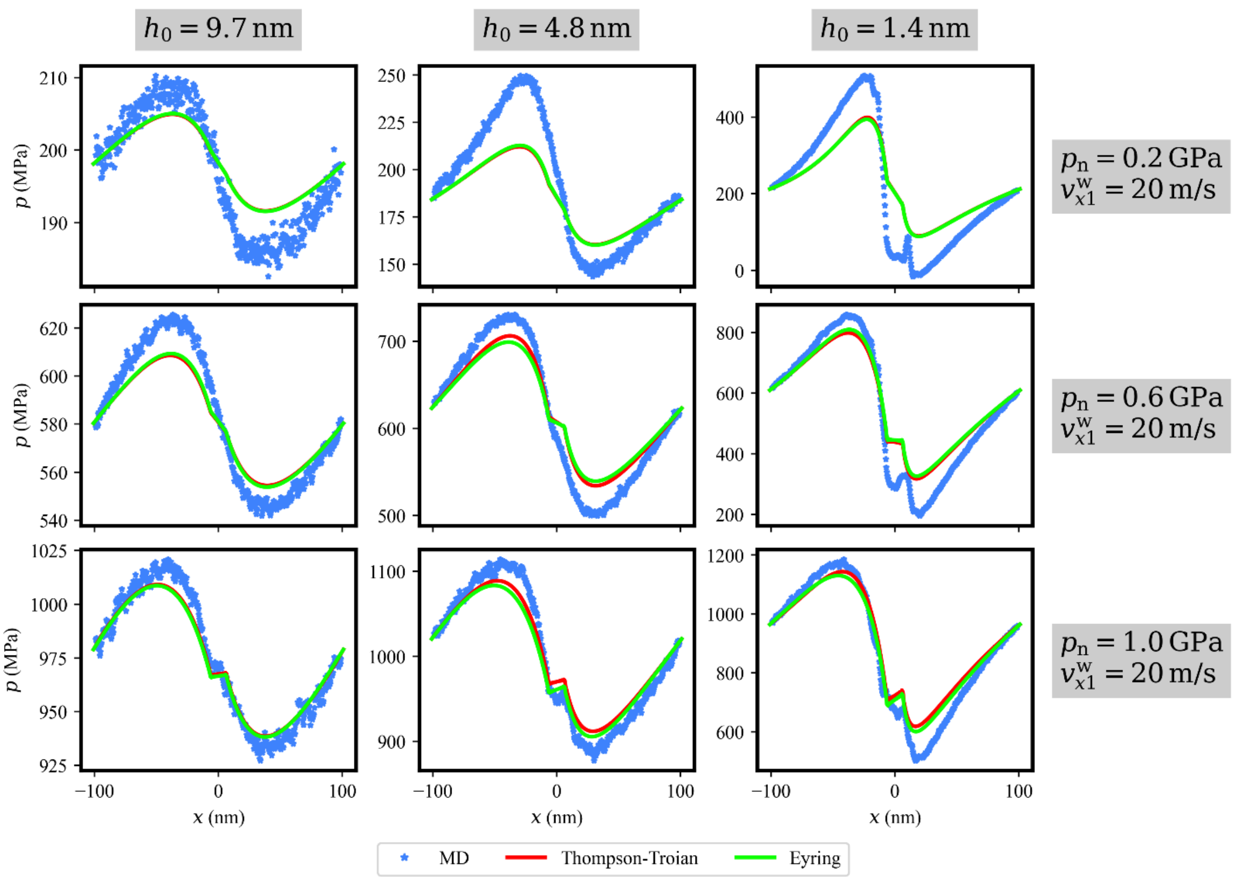

Fig. S14. Pressure profiles in the gold/hexadecane CDC for $v_{x1}^{w} = 20$ m/s and two different slip laws. $p(x)$ is shown for minimum gap heights $h_0 = [9.7, 4.8, \ 1.4]$ nm and nominal normal pressures $p_\mathrm{n} = [0.2, \ 0.6, 1.0]$ GPa. Blue stars represent the pressure $p(x)$ obtained from 10 ns steady state averages over molecular dynamics trajectories. The numerical solutions of the Reynolds equation with Thompson-Troian and Eyring slip law are displayed as red and green curves, respectively.

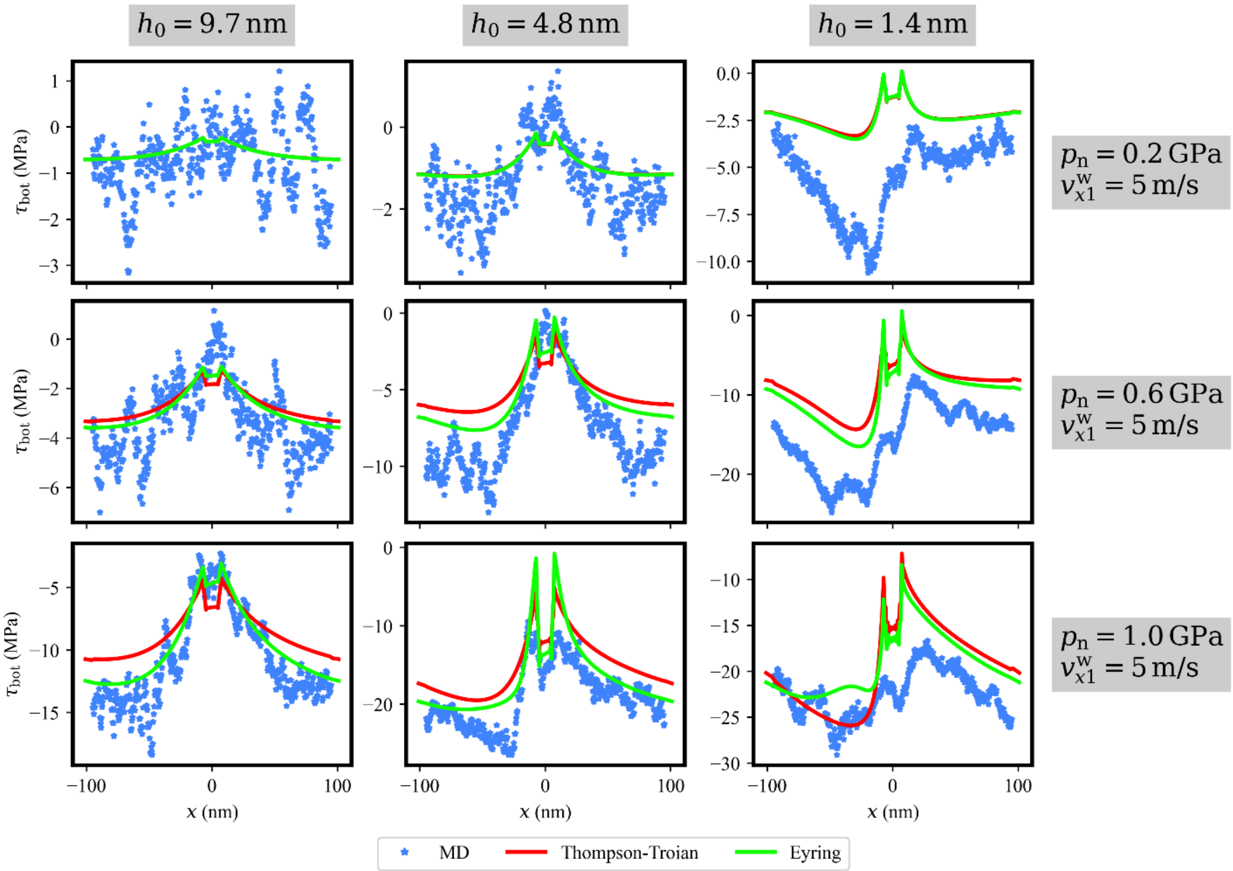

Fig. S15. Frictional stress profiles at bottom wall of the gold/hexadecane CDC for $v_{x1}^w = 5$ m/s and two different slip laws. $\tau_{bot}(x)$ is shown for minimum gap heights $h_0 = [9.7, 4.8, 1.4]$ nm and nominal normal pressures $p_n = [0.2, 0.6, 1.0]$ GPa. Blue stars represent results obtained from 10 ns steady state averages over molecular dynamics trajectories. The numerical solutions of the Reynolds equation with Thompson-Troian and Eyring slip law are displayed as red and green curves, respectively.

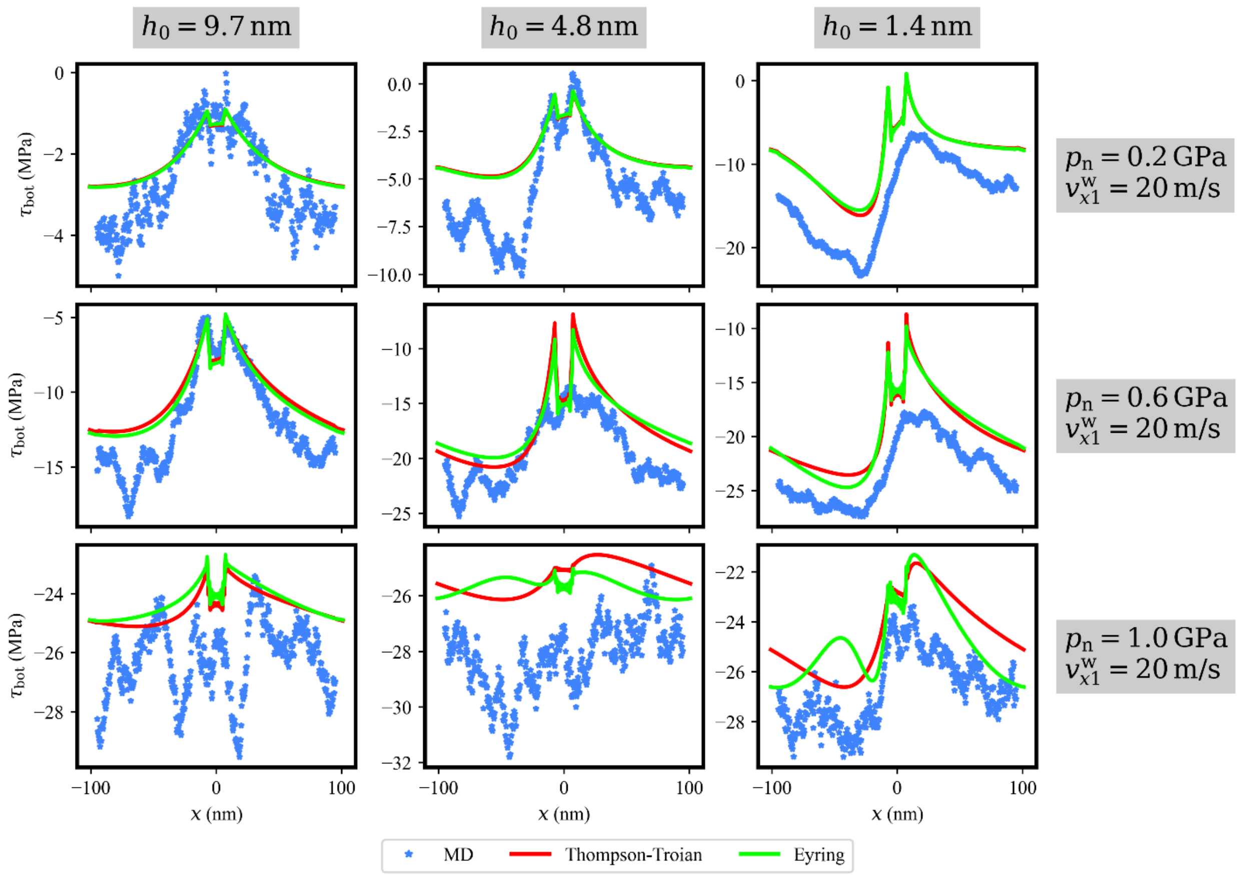

Fig. S16. Frictional stress profiles at bottom wall of the gold/hexadecane CDC for $v^w_{x1} = 20$ m/s and two different slip laws. $\tau_{bot}(x)$ is shown for minimum gap heights $h_0 = [9.7, 4.8, 1.4]$ nm and nominal normal pressures $p_\mathrm{n} = [0.2, 0.6, 1.0]$ GPa. Blue stars represent results obtained from 10 ns steady state averages over molecular dynamics trajectories. The numerical solutions of the Reynolds equation with Thompson-Troian and Eyring slip law are displayed as red and green curves, respectively.

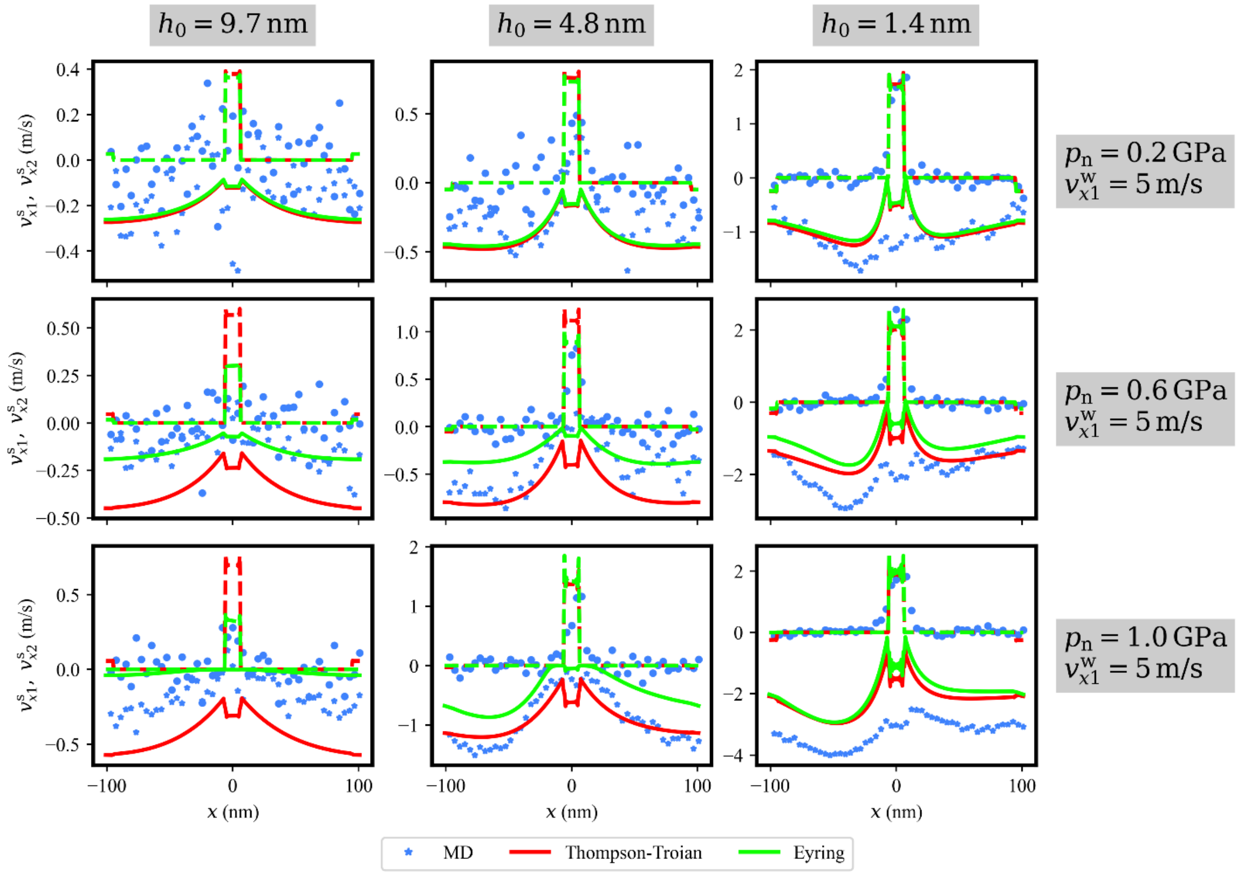

Fig. S17. Slip velocity profiles at bottom and top wall of the gold/hexadecane CDC for $v_{x1}^w =$ 5 m/s and two different slip laws. $v_{x1}^s(x)$ and v_{x2}^s are shown for minimum gap heights $h_0 = [9.7, 4.8, 1.4]$ nm and nominal normal pressures $p_n = [0.2, 0.6, 1.0]$ GPa. Blue stars represent results obtained from 10 ns steady state averages over molecular dynamics trajectories. The numerical solutions of the Reynolds equation with Thompson-Troian and Eyring slip law are displayed as red and green curves, respectively. Solid and dashed lines correspond to the bottom and top wall, respectively.

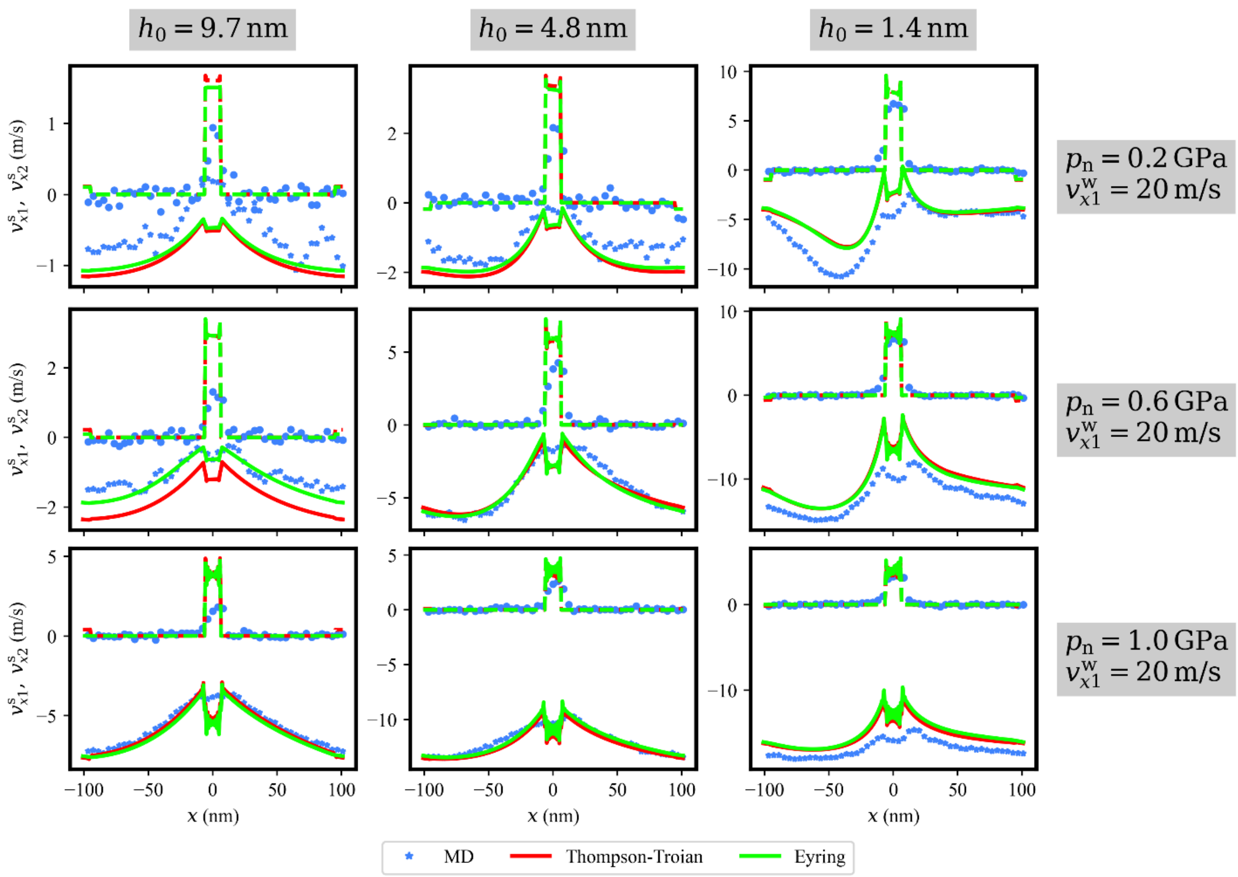

Fig. S18. Slip velocity profiles at bottom and top wall of the gold/hexadecane CDC for $v_{x1}^w = 20$ m/s and two different slip laws. $v_{x1}^s(x)$ and v_{x2}^s are shown for minimum gap heights $h_0 = [9.7, 4.8, 1.4]$ nm and nominal normal pressures $p_n = [0.2, 0.6, 1.0]$ GPa. Blue stars represent results obtained from 10 ns steady state averages over molecular dynamics trajectories. The numerical solutions of the Reynolds equation with Thompson-Troian and Eyring slip law are displayed as red and green curves, respectively. Solid and dashed lines correspond to the bottom and top wall, respectively.

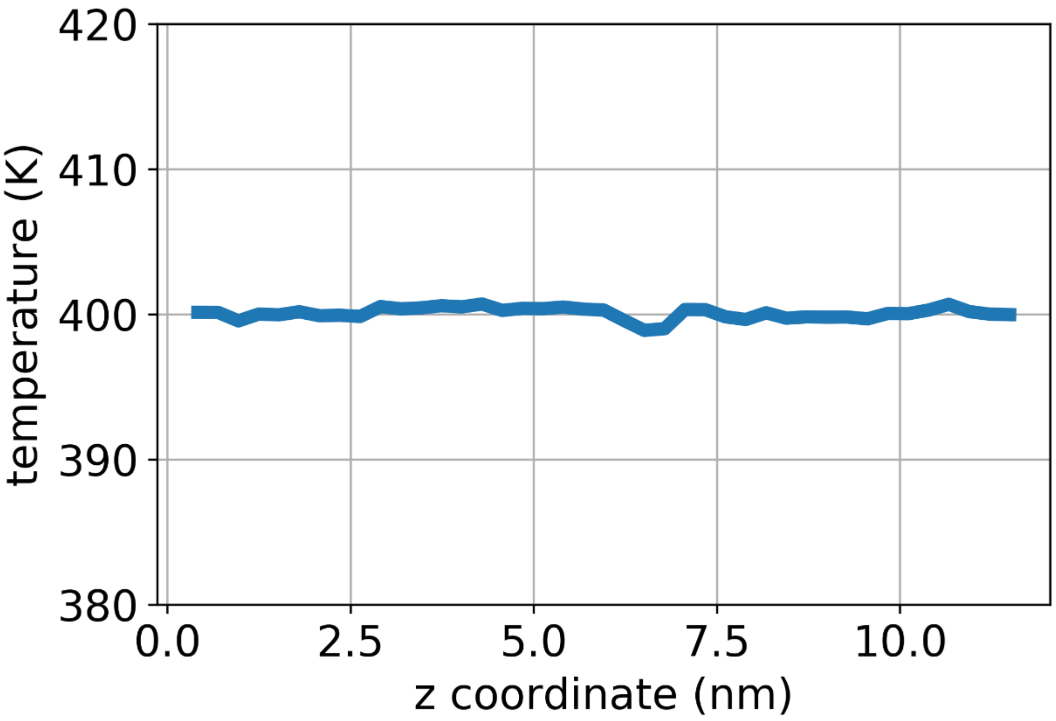

Fig. S19. Temperature profile in the parallel Au(111) channel with p_n = 1 GPa and h_0 = 2 nm and 20 m/s wall velocity. A time averaging of the local temperature for 0.5 ns in bins of size 0.28 nm has been performed. The temperature definition excludes the shearing direction. In the plot, the hexadecane lubricant is located in the zone from roughly 4.8 nm to 6.9 nm.

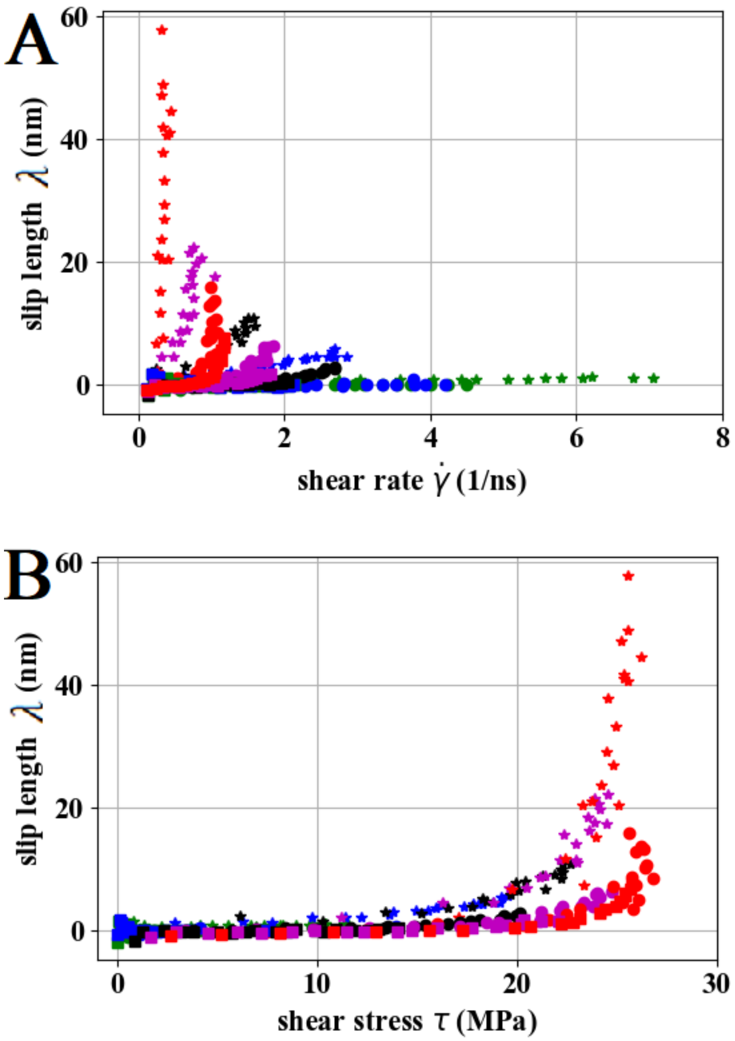

Fig. S20. Slip length λ in the hexadecane confined by the Au(111) channel with parallel flexible walls. A) Dependence of λ on shear rate $\dot{\gamma}$. B) Dependence of λ on shear stress τ. The shape of the markers represents the gap height h_0 (stars: 2 nm, squares: 5 nm, discs: 10 nm), while the colors distinguish the reference pressures p in the atomistic simulations (green: 0.1 GPa, blue: 0.4 GPa, black: 0.6 GPa, purple: 0.8 GPa, red: 1 GPa).
